# EEG Phase Can Be Predicted with Similar Accuracy across Cognitive States after Accounting for Power and Signal-to-Noise Ratio 

**DOI:** 10.1523/ENEURO.0050-23.2023

**Published:** 2023-09-01

**Authors:** Brian Kim, Brian A. Erickson, Guadalupe Fernandez-Nunez, Ryan Rich, Georgios Mentzelopoulos, Flavia Vitale, John D. Medaglia

**Affiliations:** 1 Department of Psychological and Brain Sciences, Drexel University, Philadelphia, Pennsylvania 19104; 2 Department of Bioengineering, University of Pennsylvania, Philadelphia, Pennsylvania 19104; 3 Center for Neuroengineering and Therapeutics, University of Pennsylvania, Philadelphia, Pennsylvania 19104; 4 Center for Neurotrauma, Neurodegeneration, and Restoration, Corporal Michael J. Crescenz Veterans Affairs Medical Center, Philadelphia, Pennsylvania 19104; 5 Departments of Neurology, University of Pennsylvania, Philadelphia, Pennsylvania 19104; 6 Physical Medicine and Rehabilitation, University of Pennsylvania, Philadelphia, Pennsylvania 19146; 7 Department of Neurology, Drexel University, Philadelphia, Pennsylvania 19104

**Keywords:** brain–computer interfaces, closed-loop, EEG, neural oscillations, phase, power

## Abstract

EEG phase is increasingly used in cognitive neuroscience, brain–computer interfaces, and closed-loop stimulation devices. However, it is unknown how accurate EEG phase prediction is across cognitive states. We determined the EEG phase prediction accuracy of parieto-occipital alpha waves across rest and task states in 484 participants over 11 public datasets. We were able to track EEG phase accurately across various cognitive conditions and datasets, especially during periods of high instantaneous alpha power and signal-to-noise ratio (SNR). Although resting states generally have higher accuracies than task states, absolute accuracy differences were small, with most of these differences attributable to EEG power and SNR. These results suggest that experiments and technologies using EEG phase should focus more on minimizing external noise and waiting for periods of high power rather than inducing a particular cognitive state.

## Significance Statement

EEG phase is a neural signal related to many moment-to-moment behaviors and has consequently been used to inform brain–computer interfaces and closed-loop stimulation devices. However, prior research and demonstrations have forced the user to be in a single cognitive state, such as rest, making it unclear how EEG phase can apply to the varied contexts that real individuals are placed under. The current study showed that EEG phase can be consistently well predicted across different cognitive contexts after accounting for EEG power and signal-to-noise ratio. These findings represent an important next step for both understanding the cognitive and neurobiological correlates of EEG phase and optimizing EEG-based devices to administer more effective interventions.

## Introduction

Cognition relies on cyclical processes, from recurrent cycles that occur every day (Madl et al., 2011) to cycles that occur multiple times a second to process sensory information (Blatter and Cajochen, 2007). Neural oscillations, as measured through electroencephalography (EEG), are related to these cyclical cognitive processes (Ward, 2003; Buzsáki and Draguhn, 2004). This relationship is supported by evidence that abnormal oscillations are a marker of many psychological disorders, such as depression, anxiety, and obsessive-compulsive disorder (Allen and Reznik, 2015; Thibodeau et al., 2006; Velikova et al., 2010).

A commonly used property to characterize EEG dynamics is spectral power. However, power is a time-averaged metric that commonly integrates seconds to minutes of EEG activity. Moreover, power differences are even compared over longer timescales, from seconds to years (Gao et al., 2020; Panagiotopoulou et al., 2022; Xiao et al., 2018). In contrast, many cognitive functions occur on millisecond timescales, suggesting that power fluctuations may not capture all the EEG dynamics that relate to moment-to-moment cognitive performance.

Characterizing the instantaneous properties of neural oscillations may allow us to better track cognition and behavior in real time (Lundqvist and Wutz, 2022). One instantaneous property of an oscillation is its phase, that is, the stage in its cycle at a given moment. Phase has been suggested to shape local neuronal communication by aligning the optimal output and input windows of sending and receiving neurons (Buzsáki and Wang, 2012; Canolty and Knight, 2010; Fries, 2005). As communicating neuronal populations pair to become phase locked, a predictable and consistent rhythm of communication occurs in the form of oscillations. The importance of phase coherence and activity is not limited to activity observed in local neuronal populations. It has also been demonstrated in EEG, which captures the macroscopic voltage field generated by thousands to millions of neurons (Nunez and Srinivasan, 2006). Crucially, the microscopic and macroscopic electrical activity is linked, as rhythmic EEG activity predicts local neuronal spiking (Mazzoni et al., 2010; Snyder and Smith, 2015) and cortical excitability (Bergmann et al., 2012; Massimini et al., 2003; Thies et al., 2018). As a result, the instantaneous phase of global EEG activity may mark periods of heightened corticospinal excitability and synchrony.

Accordingly, many behaviors demonstrate cyclical relationships that align with EEG phase. Single-trial analyses of EEG signals demonstrate a relationship between instantaneous phase and sensory thresholding through tactile perception (Ai and Ro, 2014) and luminance detection (Busch et al., 2009). Other studies have also demonstrated relationships between EEG phase and higher-level cognitive functions, such as attention (Dugué et al., 2016; VanRullen, 2018), decision-making (Wyart et al., 2012), and working memory (Siegel et al., 2009). Researchers have since leveraged these EEG phase–behavior relationships in closed-loop stimulation technologies (Tervo et al., 2022; Zrenner et al., 2018) and have explored EEG phase as a potential target in brain–computer interfaces (BCIs; Brunner et al., 2006; Hsu, 2015; Vigué-Guix et al., 2022).

It is important to note that EEG phase–behavior relationships are still under question as recent replication attempts have shown some null results (Keitel et al., 2022; Ruzzoli et al., 2019). Further understanding of the potential links between EEG phases and behaviors will need to come from both theoretical and empirical studies. Theoretically, postulating how brain rhythms are generated and interact with each other within the broader neurobiological context will be critical. Empirically, EEG phase–behavior experiments need stronger and more robust causal demonstrations. Closed-loop BCIs are an attractive candidate for these demonstrations because of the strong limitations they impose on phase–behavior experiments. As behavior is manipulated in real time, a closed-loop BCI must have its preprocessing parameters and features of interest chosen beforehand. Furthermore, closed-loop BCI experiments allow for precise control of stimulus presentation based on brain states, allowing us to draw stronger causal inferences in EEG phase–behavior relationships (Ramot and Martin, 2022; Vigué-Guix et al., 2022).

Real-time phase–behavior studies and phase-targeted interventions depend on accurate and precise instantaneous phase estimates. Better phase estimation improves targeting accuracy and precision, which decreases statistical variance and directly increases the statistical power of these studies (Cohen, 1998). These improvements can lead to multiple theoretical and practical benefits. Accurate targeting could causally reveal how different neuronal populations and processes interact to result in the measured phase. Additionally, these results may be used to enhance phase-dependent closed-loop neuromodulation devices that depend on EEG phase (Brunner et al., 2006; Hsu, 2015; Tervo et al., 2022; Vigué-Guix et al., 2022; Zrenner et al., 2018). Several studies have shown that accurate phase targeting depends on dynamic EEG properties, most notably the instantaneous amplitude and the signal-to-noise ratio (SNR) of the target oscillation, defined as the ratio of powers between the signal of interest and background noise (McIntosh and Sajda, 2020; Shirinpour et al., 2020; Zrenner et al., 2020). These studies consistently found that higher amplitude and SNR gave higher phase prediction accuracy, as stronger signals are easier to detect and predict. However, all these investigations were performed during one cognitive state, whether it was the resting state, or a state induced by a particular cognitive task. For phase-dependent stimulation to be deployed in a variety of contexts, it should either be robust to changes in a cognitive state or designed to account for cognitive-state related factors that may affect predictive performance.

Cognitive states can affect EEG phase prediction accuracy either through direct changes in the frequency of interest (Zrenner et al., 2020), or through indirect changes in the surrounding background noise (He, 2014; Pathania et al., 2021). Furthermore, these two factors are linked, with direct changes in one frequency band, specifically alpha, causing changes in the background excitatory, inhibitory, and high-frequency activity (Iemi et al., 2022; Peterson and Voytek, 2017). There is also evidence that different thalamic and cortical alpha generators are differentially activated and coupled under different task conditions (Halgren et al., 2019; Lundqvist and Wutz, 2022; Saalmann et al., 2012). These highly dynamic changes in EEG activity necessitate more flexible analysis tools that can account for these changes, such as phase or burst analysis (Lundqvist and Wutz, 2022). However, these tools can be further improved by directly incorporating the dynamics of cognitive state.

The most basic distinction in cognitive state is usually made between rest and task states (Buckner et al., 2008; Raichle, 2015). Cognitive rest and task states correlate with neurobiological markers. For instance, one of the oldest and most consistent observations in EEG research is the increased bursts of parieto-occipital alpha power that occur when subjects relax and close their eyes (Rusiniak et al., 2018). Alpha waves have since been associated with the inhibition of task-irrelevant brain regions (Foxe and Snyder, 2011; Klimesch, 2012). Accordingly, parieto-occipital alpha waves are a particularly useful biomarker of rest because they are easily identifiable and sensitive to changes in cognitive state (Li, 2010; Pathania et al., 2021). However, how these manifested oscillatory changes can modify phase-prediction accuracy has not been quantified.

In this work we compared EEG phase prediction accuracy in the occipital alpha band between rest and task states. To address the varied contexts in which phase prediction algorithms may be applied, we opted to analyze publicly available datasets. These datasets differed in their populations, EEG recording devices, and protocols. These inconsistencies represent some of the environmental variability that phase prediction algorithms would be exposed to in BCIs and research applications. The study used rest and task datasets obtained from two online repositories [OpenNeuro and Open Science Foundation (OSF)] and direct solicitation from researchers. The datasets used tasks aimed at a variety of cognitive domains (vigilance, executive attention, decision-making, and working memory).

We used the Educated Temporal Prediction (ETP) algorithm for phase prediction (Shirinpour et al., 2020), a parameter-free, fast, and accurate phase prediction algorithm. The ETP algorithm uses a short training period where it learns statistical properties of an individual’s EEG waveform, which are then used to make predictions. Using the predictions derived from the ETP algorithm, we compared the effect of rest and task states on EEG phase prediction accuracy. We defined accuracy as the closeness of prediction between the ground-truth waveform and the targeted phase (0°); an accuracy of one indicated that the estimated phase matched the target phase, whereas an accuracy of zero indicated that the estimated phase was at the opposite phase (180° apart).

We intended first to determine which cognitive state led to higher prediction accuracy and second to understand the underlying factors that drove changes in prediction accuracy. We hypothesized that the eyes closed resting-state would have the highest prediction accuracy, as high SNR alpha waves are the most prominent in that state. We further hypothesized that all the differences in prediction accuracy between states would be accounted for by SNR and band power changes because these two factors will be increased in the eyes closed resting-state.

## Materials and Methods

### Dataset selection procedures

#### General criteria

We conducted a systematic search for datasets in the public databases of OpenNeuro and OSF in Spring of 2021 using the search term EEG. We also obtained two datasets not available on these websites through direct solicitation and referrals. At a minimum, we required the datasets to measure scalp EEG as opposed to intracranial recordings or magnetoencephalography to ensure that we were measuring the same physical signal. We also required EEG recordings to have at least 16 channels with full head coverage arranged using the 10–20 or 10–10 international system. Although the focus of this study was on the occipital alpha frequency band, we required complete head coverage to facilitate any future experiments on different regions and frequency bands.

To be considered for the task portion of our analysis, we required that the datasets involve at least one behavioral task that required a response based on stimuli. Trials also needed clear event markers to facilitate epoching. For the resting-state datasets, participants needed to be idle with either their eyes closed or eyes opened while relaxed and fixated on an object in front of them.

#### Cognitive domain criteria

We included tasks using various cognitive domains. We categorized each task based on the main cognitive function its conditions were meant to manipulate. Although we did not include these categories in our statistical model, we were interested in including a broad variety of task data. We classified each task as follows.

##### Vigilance

To measure vigilance, we looked for datasets with a psychomotor vigilance task (PVT), a reaction-time-based task that measures participants’ speed in detecting a visual stimulus. We included only PVT datasets that used a simple alerting stimulus (no letter stimuli that used language faculties or competing stimuli that required choices). A representative example of a vigilance task comes from Wong et al.’s (2018) experiment in which participants stared at a fixation cross and reacted as quickly as possible to the appearance of a millisecond counter. A key feature of the PVT task is jittering of the interstimulus interval to prevent anticipation effects (the interstimulus interval was between 2 and 10 s).

##### Executive attention

Executive attention tasks are typically rule based and require individuals to select a correct response among distractors. A representative example of an executive attention task comes from Foster et al.’s (2021) experiment. In the study, participants were initially alerted to a trial with a fixation dot. A cue was then presented, which indicated the location of subsequent bull’s-eyes. Depending on the condition, patients were to covertly monitor this cued location for a contrast change in the bull’s-eyes or overtly monitor the central fixation for a decrease in contrast in the fixation dot.

##### Decision-making

Decision-making tasks force users to make decisions under uncertainty. The uncertainty can arise from stimulus identity (perceptual uncertainty) or from uncertain outcomes (outcome uncertainty). The current project included dataset(s) testing the latter, where participants were expected to learn the statistical properties of their decision outcomes throughout a task. A representative example of a decision-making task comes from Cavanagh’s (2015) experiment, which consisted of three slot machines whose payouts were scheduled periodically, so that at any one time, a single slot machine would have the best payout. Participants were expected to learn the reward schedule and choose the slot machines that maximized their payout.

##### Working memory

Working memory tasks involve storing and manipulating information internally. The current project included datasets testing visuospatial working memory, where participants stored and made decisions based on visual information about a stimulus. A representative example of a working memory task comes from Oh et al.’s (2019) experiment in which participants were shown 20 bars, all oriented in the same direction or in varying orientations. After a 1500 ms retention interval, participants saw a test probe and determined whether it was previously present.

##### Datasets

We obtained a total of 11 datasets. Across all datasets, we had 578 participants. For the resting-state condition, we had a total of 436 participants. For the task set condition, we had a total of 178 participants. The 36 participants from the vigilance task appeared in both task and rest datasets.

After excluding some datasets during our EEG preprocessing steps (see below, EEG preprocessing), we had 484 participants and 1,518, 674 EEG predictions (156,512 eyes closed condition, 170,528 eyes open condition; 1191,634 task condition). A full list of the datasets is provided in Tables 1 and 2.

**Table 1:** Description of task datasets used along with number of participants used for the initial analysis

Title of Paper	Abbreviation used in this article	Source	Cognitive domain	Short description of task	Duration of recordings	Number of participants included for analysis
Positive effects of mindfulness-based training on energy maintenance and the eeg correlates of sustained attention in a cohort of nurses (Wong et al., 2018)	PVT	https://osf.io/y23k8/	Perception	Participants stare at a fixation cross and respond to a millisecond counter.	20 min block	36
Real-time EEG feedback on alpha power lateralization leads to behavioral improvements in a covert attention task (Schneider et al., 2020)	ALPH	https://openneuro.org/datasets/ds002034/versions/1.0.3	Attention	Participants stare at a central fixation while covertly attending to a circle with an inscribed cross. Participants react to the disappearance of the inscribed cross.	40 min block	14
Covert attention increases the gain of stimulus evoked population codes (Foster et al., 2021)	COV	https://osf.io/hmvzc/	Attention	Participants monitor a target bull’s-eye and check for a decrease in contrast.	Approximately three sessions of 60 min on task	24
No evidence that spontaneous eye blink rate can predict baseline attentional blink size, or the effects of tDCS on attentional blink size (Reteig et al., 2022)	AB	https://openneuro.org/datasets/ds001810/versions/1.1.0	Attention	Participants engaged in an attentional blink paradigm, where a second stimulus appears shortly after a first one, and they were asked to identify both stimuli.	Three 20 min blocks	47
Cortical δ activity reflects reward prediction error and related behavioral adjustments, but at different times (Cavanagh, 2015)	B3	https://openneuro.org/datasets/ds003458/versions/1.1.0	Decision-making	Participants are expected to learn the payoff structure of three different slot machines to maximize rewards. Probabilities of each machine are modeled by a sine wave.	40 min blocks	23
Ensemble representations reveal distinct neural coding of visual working memory (Oh et al., 2019)	ENS	https://osf.io/nvaz3/	Working memory	Participants viewed twenty bars 20 judged whether a probe bar was present. In the second experiment, participants were to recall the cued location and adjust the probe to the cued orientation.	Approximately 100 min on task	34

**Table 2:** Description of rest datasets used along with number of participants used for the initial analysis

Title of paper	Abbreviation used in current paper	Source	Type of resting-state condition recorded	Number of participants included for analysis
Positive effects of mindfulness-based training on energy maintenance and the eeg correlates of sustained attention in a cohort of nurses (Wong et al., 2018)	PVT	https://osf.io/y23k8/	4 min of eyes-open rest 4 min of eyes-closed rest 4 min of eyes-open rest (after experimental intervention)	36
Weak rTMS-induced electric fields produce neural entrainment in humans (Zmeykina et al., 2019)	TMS	https://osf.io/3xuk8/	4 min of eyes-open rest 4 min of eyes-closed rest	23
Heterogeneity of EEG resting-state brain networks in absolute pitch (Greber et al., 2020)	ABS	https://osf.io/hbz28/	3 min of eyes-closed rest	104
EEG resting activity in highly sensitive and non-highly sensitive persons (Dimulescu et al., 2020)	SENS	https://osf.io/pgtu6/	15 min of eyes-closed rest	20
Dual-process contributions to creativity in jazz improvisations: An SPM-EEG study (Rosen et al., 2020)	JAZZ	Solicitation	2 min of eyes-open rest 2 min of eyes-closed rest	38
Within and between-person correlates of the temporal dynamics of resting EEG microstates (Zanesco et al., 2020)	MICRO	https://ftp.gwdg.de/pub/misc/MPI-Leipzig_Mind-Brain-Body-LEMON/	8 min of eyes-open rest 8 min of eyes-closed rest	215

### Educated temporal prediction algorithm

An important factor in phase prediction is the speed of the algorithm because real-time algorithms have limited time to complete computations. Prior methods involve forward prediction in the time domain using autoregressive methods (Zrenner et al., 2018) or in the frequency domain using Fourier-based approaches (Mansouri et al., 2017). The current analysis used the Educated Temporal Prediction (ETP) Algorithm (Shirinpour et al., 2020) to predict EEG phase. Unlike autoregressive models, ETP is parameter free and does not require as much computational power. Fourier-based approaches also do not require many parameters and are computationally faster than autoregressive models, but they assume periodicity and harmonicity of the underlying signal. The ETP algorithm (Shirinpour et al., 2020) obviates these limitations by using a short training period where it learns statistical properties of an individual’s EEG waveform. The algorithm requires minimal assumptions about the underlying signal and has been shown to perform more quickly and accurately than the other approaches. A very similar approach has been used by Vigué-Guix et al. (2022) in a real-time closed-loop BCI context that performed similarly to other estimate approaches. The main difference in their approach was basing predictions on the individual alpha frequency instead of the average interpeak interval, the distance between consecutive peaks.

**Figure 1.**
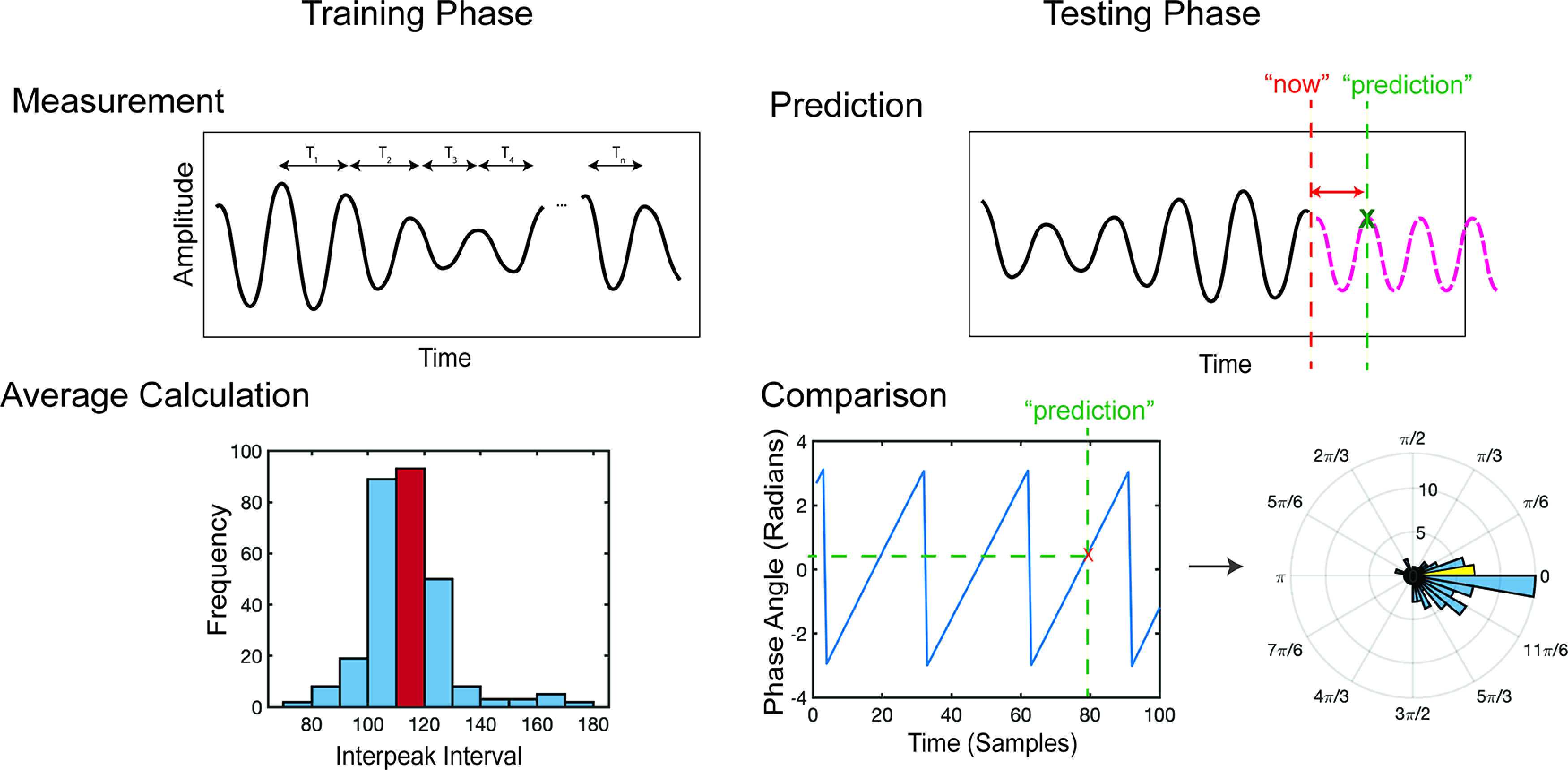
Diagram of the ETP algorithm. Left, The first phase is a training phase, where the average interpeak interval is learned from resting-state data. Right, In the testing phase, this average interpeak interval can be added to the last seen peak to predict future peaks. We can then compare ground-truth angles computed from the Hilbert transform with the expected angle. The phase angle of a peak corresponds to 0°, so we expect predicted phase angles to be close to zero as indicated by the polar histogram.

The first stage of the ETP algorithm is a training phase (Fig. 1). In the training phase, the raw time series data are bandpass filtered to the alpha range (8–13 Hz) by a Hamming-windowed finite impulse response filter (FIR; see below, EEG preprocessing). This filtered time series is used to compute the interpeak interval, which was used to predict future peaks. Consequently, we split each dataset into two parts (see below, Epoch extraction), with one part used to learn the average interpeak interval (training data) and the other part used to test this interval for prediction (test data).

The second phase of ETP is the test phase. In the test phase, a 500 ms window is slid over the test data time point by time point. Each window of data are filtered to the alpha range via a Brickwall filter. Local maxima are designated as peaks in this window. If no peaks are found, the algorithm moves on to the next window. The latency of the next peak is then predicted as the time of the last seen peak plus the average interpeak interval learned from the training data.

Once the whole dataset has run through ETP, a comparison ground-truth version of the time series is computed to establish the accuracy of the predictions. Like the procedure in the ETP training phase, the test time series is filtered in the alpha range via a Hamming-windowed FIR filter. The instantaneous phase of the filtered time series is then extracted via a Hilbert transform. Comparing the times predicted as peaks (0°) by ETP to the ground-truth instantaneous phase at those times results in an accuracy distribution.

Although ETP originally learned the average interpeak interval from separately recorded resting-state data, we opted to use intertrial intervals to train the ETP algorithm for our task datasets because all but one task dataset did not have corresponding resting-state data. Accordingly, all our task datasets were epoched to separate intertrial intervals from on-trial intervals.

**Figure 2.**
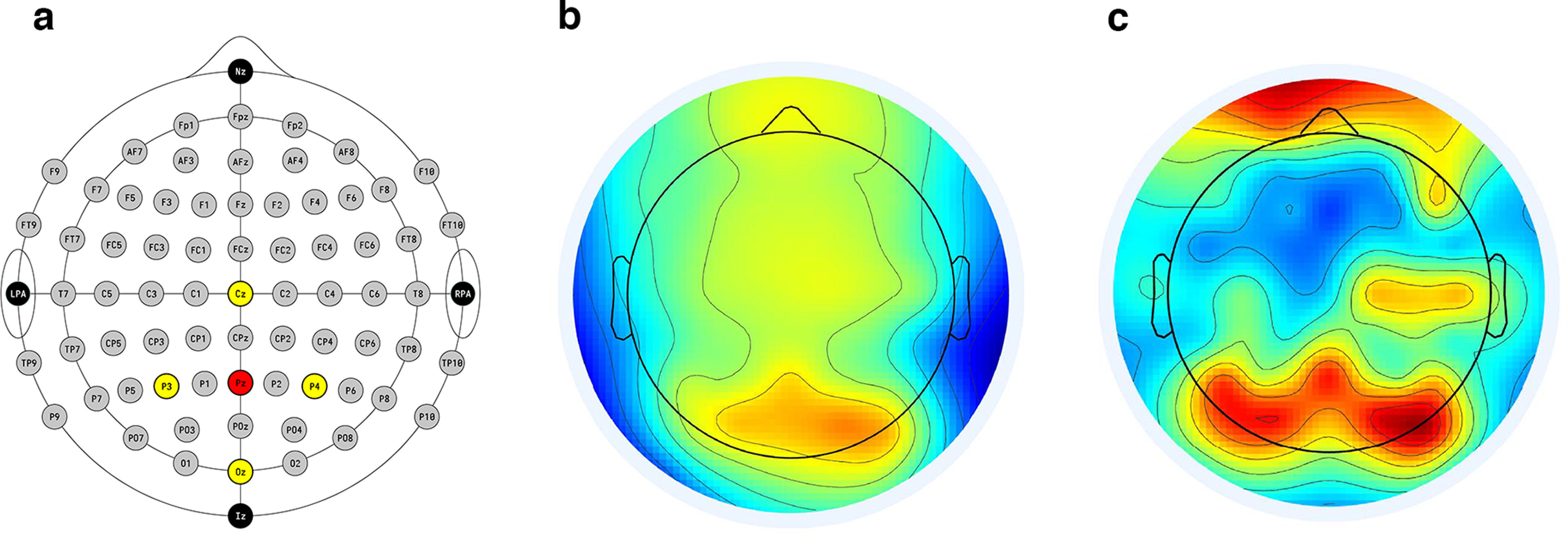
EEG electrode placement and surface Laplacian. ***a***, Diagram of the 10–10 system used in the current analysis. The electrodes used in the Laplacian montage are colored with the central electrode (Pz) in red, and the surrounding electrodes (Oz, Cz, P4, and P3) in yellow. ***b***, ***c***, Applying the Laplacian montage allows us to see higher spatial frequencies. Scalp topographies in the alpha band (8–13 Hz) are displayed without (***b***) and with (***c***) the Laplacian montage applied.

We used the publicly available version of the ETP algorithm (Shirinpour et al., 2020) with slight modifications to use individual peak alpha frequencies (described below) and to run on epoched datasets. (The original algorithm expects continuous data.) Although we wrote our main EEG preprocessing suite in EEGLAB software, any additional filtering used by the ETP algorithm used the Fieldtrip toolbox (Oostenveld et al., 2011), version 20201214, to ensure consistency with the original publicly available version of the ETP algorithm. The ETP algorithm also applies spatial filtering via the Laplacian montage (Gordon and Rzempoluck, 2004), using this spatially filtered signal for prediction. For our Laplacian montage, we chose to center on electrode Pz, with surrounding electrodes Oz, Cz, P4, and P3 as parieto-occipital electrodes exhibit the highest alpha power (Fig. 2).

### Dataset curation procedures

All EEG preprocessing was done using EEGLAB version 2021.0 (Delorme and Makeig, 2004) on MATLAB R2020b (MathWorks).

#### EEG preprocessing

EEG preprocessing methods can significantly alter the results (Luck, 2014). To ensure consistency in our preprocessing steps, we included only datasets we could gain access to, either DC recordings or recordings with only a wideband filter (with a low-pass filter no lower than 60 Hz) and/or notch filter applied (at 50 Hz or 60 Hz). Datasets with any other preprocessing steps already applied were excluded.

To enable fair comparisons, we downsampled all datasets to the lowest sampling rate in the set (250 Hz) and included only the channel locations common across all the datasets (the 32 standard channels of the 10–20 system). Before ETP analysis we applied an additional Hamming window FIR bandpass from 0.15 to 60 Hz to ensure all datasets contained the same frequency band and to remove DC drift. We did not apply an additional notch filter, as line noise frequencies (50 and 60 Hz) are significantly above our frequency band of interest.

### Bad channel and artifact rejection

Bad channels usually occur because of poor connections between the electrode and scalp. Bad channel rejection is a common step in preprocessing pipelines (Bigdely-Shamlo et al., 2015; Kothe and Makeig, 2013).

Therefore, we included an automatic bad channel rejection algorithm in our pipeline, implemented by the clean_rawdata plug-in in EEGLAB, which uses methods from the BCILAB toolbox (Kothe and Makeig, 2013). We used the default settings to remove channels that (1) had a correlation <0.85 to a reconstruction of itself based on surrounding channels, (2) had high-frequency noise 4 SDs above the total channel population, and (3) had flat data for >5 s. Although 4 SDs above the population mean is aggressive, it is consistent with the default settings of the clean_rawdata plug-in and has precedence in the BCI literature and other preprocessing pipelines that use 4 or 5 SDs (Bigdely-Shamlo et al., 2015; Chang et al., 2020). We also excluded channels that were not related to brain signals, such as electrooculogram and trigger channels. To reject transient artifacts, we removed epochs that contained an amplitude exceeding three times the SD of the dataset.

#### Measuring signal-to-noise ratio and individual alpha frequency

Environmental and intersubject variability can significantly alter brain behavior, which can manifest as differences in EEG signals (Farzan et al., 2017). To quantitatively model differences in signal quality across recordings, we decided to include SNR in our statistical models. We used the approach of Zrenner et al. (2020) in computing SNR because of its simplicity and focus on real-time phase estimation. We conducted spectral analysis of the whole recording using Welch’s method (Welch, 1967) (50% overlapping epochs of 2 s duration, linearly detrended, Hann windowed, Fourier transformed, and averaged). We then determined the frequency in the range of 8 and 13 Hz with the highest power, which was designated as the individual alpha frequency (IAF). In the case of more than one spectral peak in the 8 and 13 Hz range, we designated the IAF as the average of the peak frequencies weighted by their alpha power (Klimesch, 1999). After converting the power spectra into log-log space, low-frequency 1/f noise was estimated by fitting a straight line using points outside our targeted frequency range (0.5–7 and 35–65 Hz). SNR was defined as the difference between the amplitude at the IAF and the value of the fitted 1/f at the IAF on the log scale in units of decibels.

An additional benefit of this calculation is the ability to model individual variability in peak alpha frequency, which differs significantly across individuals (Klimesch, 1999). The original ETP algorithm used an alpha bandpass filter from 8 to 13 Hz, which assumed a peak alpha frequency of 10.5 Hz for all participants. As using individualized alpha frequencies has shown improvements in model prediction performance and stimulation effects (Klimesch et al., 2003; Thomas and Vinod, 2016), we opted to base our filters on individualized peak alpha frequencies instead of the nonspecific 10.5 Hz frequency.

We were further able to improve signal quality by using SNR and IAF as exclusion criteria. We removed all recordings with a negative SNR, specifically those recordings with a higher 1/f noise level than alpha power at the peak frequency. We also removed all recordings where no spectral peak could be determined. We removed a participant from analysis when none of their recordings passed our criteria. Of our original 578 participants, 94 were excluded, leaving 484 participants for the subsequent analyses.

#### Epoch extraction

We were interested in three types of epochs—resting intervals, on-task intervals, and intertrial intervals. Although we were primarily concerned with phase prediction accuracy between rest and task, we included intertrial intervals for two reasons. First, our phase prediction algorithm requires rest data to learn the average interpeak interval, but most of the task datasets did not provide pure resting-state data. Using intertrial intervals as a substitute for resting state allowed us to greatly expand the scope of our study. Second, although not part of the main analyses, intertrial intervals constitute a significant portion of on-task time and may warrant further analysis.

For each task dataset, we defined two sets of epochs. On-task epochs were extracted starting at the beginning of each trial up to the end of a trial (e.g., after a response or after the window for responding had closed). Intertrial or pseudo-rest epochs were extracted immediately after the end of a trial up to the beginning of the next trial. We used pseudo-rest epochs as the training set to learn the average interpeak interval of an individual. On-task epochs were used as the testing set to estimate EEG phase prediction accuracy. Before artifact rejection, the number of epochs in each of these sets was equal. The length of the pseudo-rest epochs ranged from 250 ms to 2 s, whereas the length of the on-task epochs ranged from 1 to 7.1 s.

For each resting-state dataset, we split the data into half, with the first half designated as the training set and the second half designated as the testing set. Each resting-state set was then split into epochs of a length of 2 s, matching the shortest on-task epoch length from our task datasets.

#### Statistical procedure

At each window step, the ETP algorithm returns a prediction for the timing of the next peak. We compared these predictions with the ground-truth waveform and computed their accuracies. We defined accuracy as the closeness of prediction in the ground-truth waveform to the targeted phase (0°) as follows:

$$Accuracy\;=\;1-\frac{1}{180}\;\left|\theta_{i}\;-\;\theta_{t}\right|,$$

where θ_i_ is the estimated phase for trial *i*, and θ_t_ is the targeted phase. An accuracy of one indicates that the estimated phase matched the target phase, whereas an accuracy of zero indicates that the estimated phase was at the opposite phase (180° apart).

Importantly, these predictions are part of a nested structure. Each peak is part of a trial, which is part of an experimental block, which is conducted on an individual, which is part of a dataset, which is separated into resting and task states. As a result of this nesting, the assumption of independence is violated, making a standard linear regression inappropriate (James et al., 2021). We used multilevel modeling with maximum-likelihood estimation to account for this hierarchical structure (Raudenbush and Bryk, 2002). A multilevel modeling approach allowed us to model the variances for hierarchical confounding variables as random effects and measure the variances of the condition of interest as fixed effects (Meteyard and Davies, 2020). Multilevel modeling also does not require sphericity, as it models heteroskedasticity as needed (Rosopa et al., 2013).

We used the following model:

accuracy ∼ state + (1 | dataset_id) + (1 | dataset_id : individual_id),

where *accuracy* represents the accuracy of a trial, *state* is a categorical variable indicating whether the individual is in a eyes-open resting-state, eyes-closed resting-state or task state, and *dataset_id* and *individual_id* are categorical variables indicating the dataset and individual from which the measurement was taken. The nested random effect of *dataset_id: individual_id* was included because of how we coded our variables. Specifically, the first individual in each dataset was given an *individual_id* of one, and then two, and so on. The nested random effect of *dataset_id: individual_id* allowed us to distinguish an individual with the *id* of one as belonging to a particular dataset.

We only considered random intercepts for the dataset and individual. We did not consider random slopes as each dataset and individual were part of only a rest or task dataset. Although there was a single dataset that contained both rest and task data (Wong et al., 2018), we decided to model the rest and task data as distinct datasets with different individuals. This simplification was made to promote consistency with the other datasets that only had recordings of a single type and to reduce the complexity of our linear models.

High-signal band power is critical for reliable phase estimations as higher power gives higher confidence in EEG measurements. As a result, any differences in EEG phase prediction accuracy may be partially explained by higher signal power. Our secondary analysis included occipital alpha power as a covariate to explain how cognitive domain may affect EEG predicted phase accuracy. To measure power, we took the instantaneous power computed from the Hilbert transform at the moment of prediction and included it as a covariate. We used the following intermediate model to identify the effects of rest or task on phase prediction accuracy with alpha power as a covariate:

accuracy ∼ state + alpha_ power + state:alpha_ power + (alpha_power | dataset_ id) + (alpha_power | dataset_ id : individual_ id).

In addition to this instantaneous measure of power, we also included the SNR over the whole recording. We used the following maximal model (Barr et al., 2013) to identify the effects of rest or task on phase prediction accuracy with alpha power and overall SNR as a covariate:

accuracy ∼ state + alpha_ power + SNR + state : SNR + state : alpha_ power + SNR : alpha_ power + state : alpha_ power : SNR + (alpha_ power*SNR | dataset_ id) + (alpha_ power*SNR | dataset_ id : individual_ id),

where *SNR* represents the signal-to-noise ratio calculated over the entire recording, and α*_power* represents the instantaneous alpha power. In this model, we included both random intercepts and slopes for alpha power and SNR, as subjects and datasets are likely to have different baselines and effects of alpha power and SNR on accuracy.

We conducted multilevel modeling in MATLAB R2020b, using the *fitlme* function. We categorized regression terms with a *p* value < 0.05 as significant.

### Data availability

The data that support the findings of this study are listed in Tables 1 and 2. The table contains links to public repositories that contain the data. Only one dataset listed in the table does not have a publicly available link, which has been obtained by direct solicitation from the corresponding author. The code that supports the findings of this study are available on GitHub under the following link: https://github.com/CogNeW/project_eeg_public_dataset.

## Results

### Descriptive statistics of EEG signals

**Figure 3.**
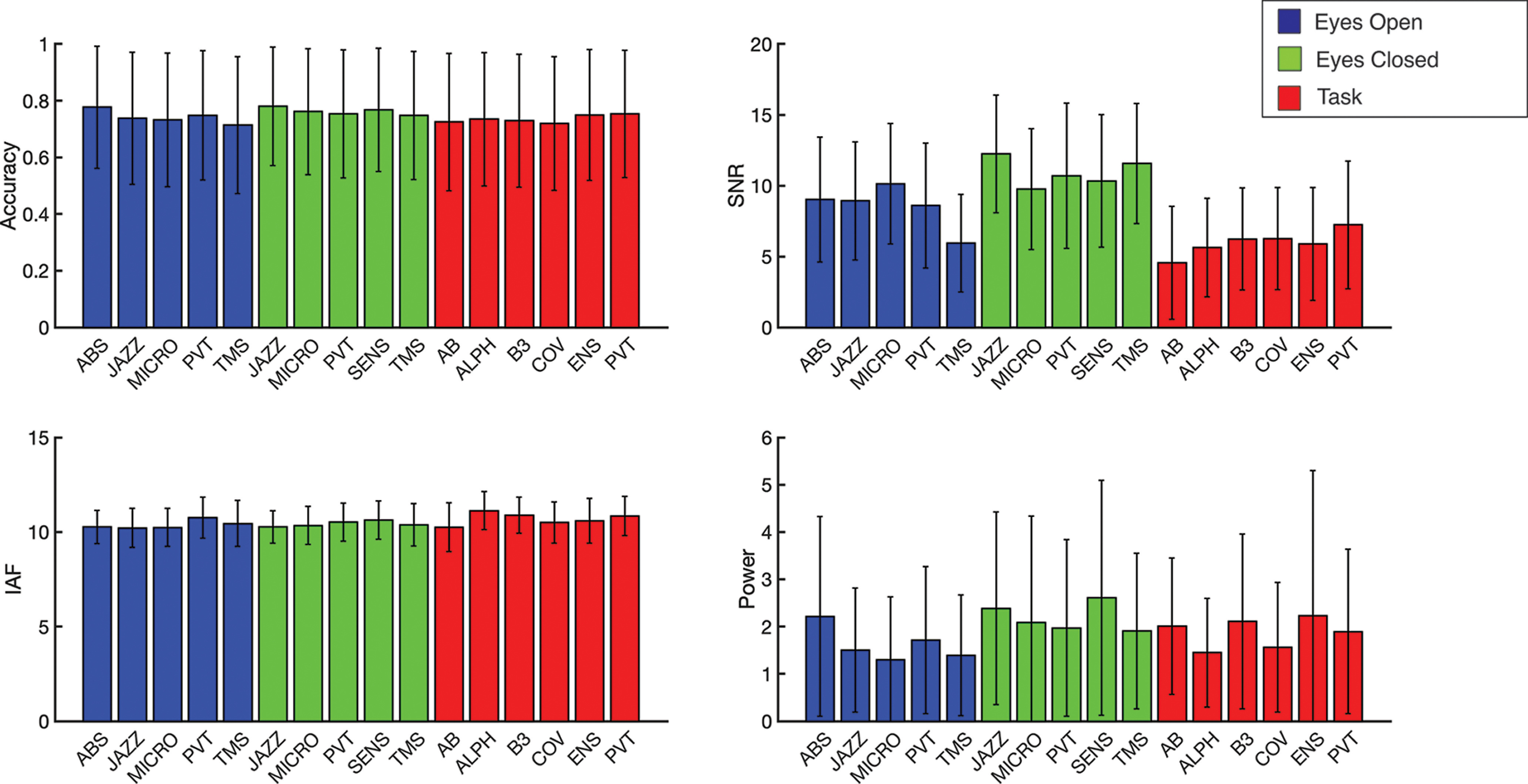
Accuracy, SNR, IAF, and instantaneous power distributions across datasets. The height of each bar represents the average value, and the error bars represent 1 SD. Bars are filled based on which cognitive state they represent. Abbreviations are used for each dataset name. Table 1 contains the full dataset name and references.

Across all the predictions made by the algorithm on the datasets, the average phase prediction accuracy was 73.70% (SD = 23.40%). However, accuracy only describes the spread of the predictions. In terms of phase angles, our algorithm was on average +6.30° away from the target (SD = 47.34°). In the context of a 10 Hz alpha wave, our predictions were 3.5 ms late (SD = 13.15 ms). These results align with previous attempts at phase targeting (Madsen et al., 2021; Vigué-Guix et al., 2022; Zrenner et al., 2018), whose procedures had a mean error of −12° to 5°, with SDs from 25° to 55°. The average instantaneous power was 1.91 μV/Hz (SD = 2.06 μV/Hz). The average SNR was 6.59 (SD = 4.37). Split across conditions, the eyes-closed (EC) rest state showed the highest average instantaneous band power (mean = 2.18 μV/Hz, SD = 2.20 μV/Hz), followed by the task state (mean = 1.93 μV/Hz, SD = 2.10 μV/Hz), and then the eyes-open (EO) rest state (mean = 1.57 μV/Hz, SD = 1.59 μV/Hz). Furthermore, the EC rest state had the highest average SNR (mean = 10.36 dB, SD = 4.57 dB), followed by the EO rest state (mean = 9.35 dB, SD = 4.39 dB), and then the task state (mean = 5.71 dB, SD = 3.89 dB). Complete metrics by dataset are provided in Figure 3.

### Cognitive status affects EEG phase prediction accuracy

Our basic model indicated that cognitive status affected EEG phase prediction accuracy. The baseline category was EC rest, which had a phase prediction accuracy of 76.3%, 95% CI [74.9, 77.7%] The EO rest state reduced accuracy by 2.03% (*t*_(1.51*10^6^)_ = −2.10; *p* = 0.035) 95% CI [0.139, 3.92%]. The task state also reduced accuracy by 2.78% (*t*_(1.51*10^6^)_ = −2.91; *p* = 0.0036), 95% CI [0.90%, 4.65%]. A likelihood ratio test indicated that this basic model explained the data better than a null model [χ^2^(2) = 34842, *p* < 1e-5]. These results support our hypothesis that the eyes closed resting-state would have the highest prediction accuracy.

### Differences in prediction accuracy remain after accounting for instantaneous power

When including just power as a covariate, the baseline accuracy of EC rest dropped to 64.94%, 95% CI [63.4%, 66.4%]. Power had a significant effect on accuracy (*t*_(1.51*10^6^)_ = 9.98; *p* < 1e-5). A unit increase in instantaneous power led to a 6.80% increase in accuracy, 95% CI [5.46%, 8.13%] (Fig. 3). After accounting for power, the EO rest state did not significantly affect accuracy (*t*_(1.51*10^6^)_ = −1.66; *p* = 0.10). However, there was a significant interaction of the EO rest state and power (*t*_(1.51*10^6^)_ = 2.17; *p* = 0.03), with an effect size of 2.06%, 95% CI [0.20%, 3.92%]. After accounting for power, the task state still had a significant effect on accuracy (*t*_(1.51*10^6^)_ = −2.17; *p* = 0.03), with an effect size of −2.27%, 95% CI [−0.22%, −4.32%]. However, there was no significant interaction between the task state and power (*t*_(1.51*10^6^)_ = 0.52; *p* = 0.60). These results do not align with our hypothesis that differences in phase prediction accuracy because of cognitive states do not persist after accounting for instantaneous power. A likelihood ratio test indicated that this model explained the data better than the previous model [χ^2^(7) = 160680, *p* < 1e-5].

### Including SNR and power accounts for all differences among cognitive states

When including power and SNR as covariates, the baseline accuracy of EC rest dropped to 59.56%, 95% CI [57.3%, 61.9%]. The EO rest state did not significantly affect accuracy (*t*_(1.51*10^6^)_ = −0.90; *p* = 0.37), whereas being in a task state was also not significant (*t*_(1.51*10^6^)_ = −0.61; *p* = 0.54). These results align with our hypothesis that differences in phase prediction accuracy because of cognitive states do not persist after accounting for instantaneous power and SNR.

**Figure 4.**
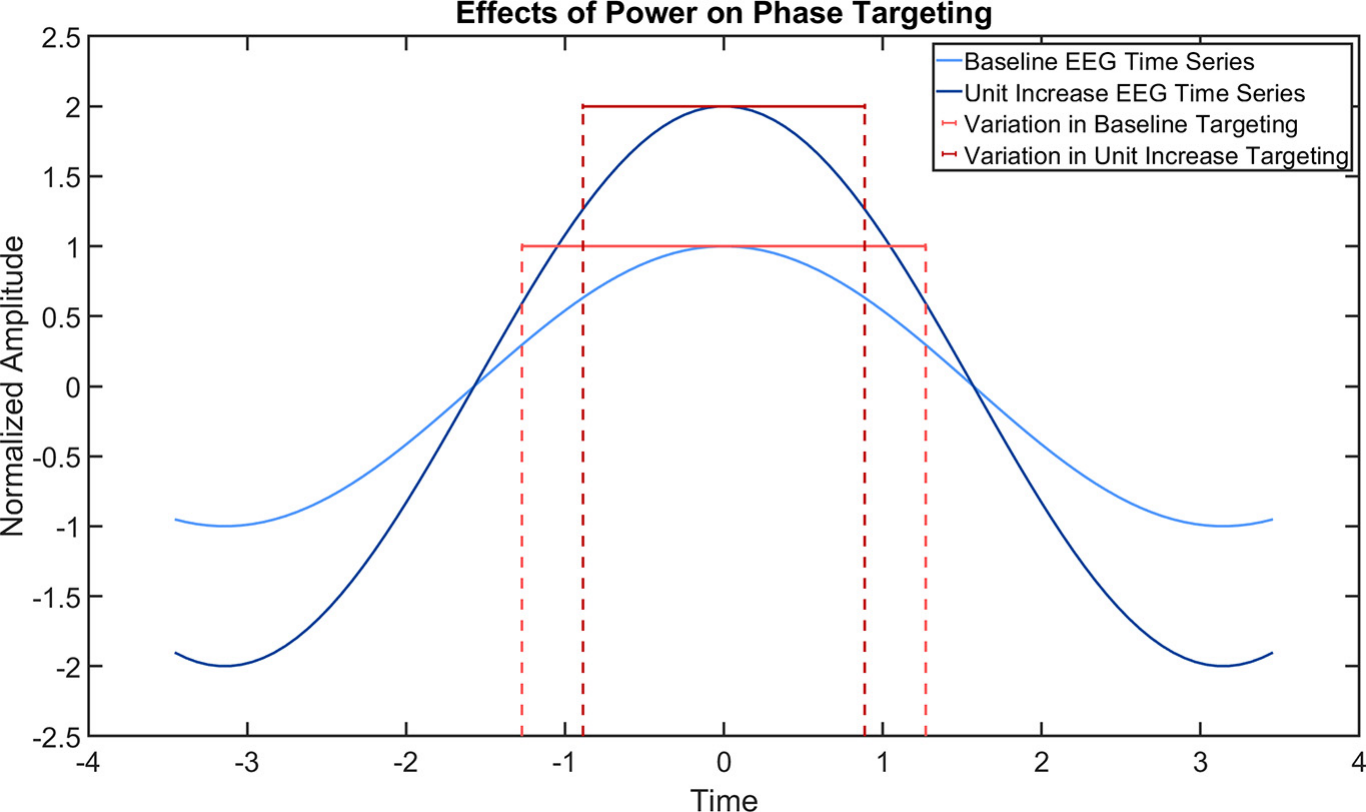
The change in phase prediction accuracy because of a unit increase in power. The light blue curve represents an idealized EEG time series with amplitude 1, whereas the darker blue curve represents a time series with a unit increase in amplitude. The red error bars represent the average spread in peak prediction. Notice that the unit increase in power has a tighter distribution by 12.23% than the baseline time series.

Accordingly, power had a significant effect on accuracy (*t*_(1.51*10^6^)_ = 8.47; *p* < 1e-5). A unit increase in instantaneous power led to a 12.23% increase in accuracy, 95% CI [9.40%, 15.06%] (Fig. 4). SNR also had a significant effect on accuracy (*t*_(1.51*10^6^)_ = 4.40; *p* = 0.00,001), with a unit increase in power leading to a 0.47% increase in accuracy, 95% CI [0.26%, 0.68%]. There was a significant interaction between power and SNR (*t*_(1.51*10^6^)_ = −5.29; *p* < 1e-5), with an effect size of −0.49%, 95% CI [−0.31%, −0.67%]. These results align with our hypothesis that most of the accuracy differences between cognitive states arose from instantaneous power and SNR. A likelihood ratio test indicated that this model explained the data better than the previous model [χ^2^(20) = 9182, *p* < 1e-5]. Follow-up contrasts indicated no further differences in phase prediction accuracy among conditions and no differences in the effects of power and SNR on phase prediction accuracy among conditions. Full regression results are shown in Table 3.

**Table 3:** Regression results for the full model

Variable	Coefficient	SE	*t* Statistic	Probability
**Intercept (state EC)**	**59.56%**	**1.17%**	**50.83**	**<1e-5**
**Power**	**12.23%**	**1.44%**	**8.47**	**<1e-5**
Status EO	−1.42%	1.58%	−0.90	0.37
Status task	−0.94%	1.52%	−0.62	0.54
**SNR**	**0.47%**	**0.11%**	**4.40**	**0.00001**
Power:StatusEO	1.90%	1.99%	0.96	0.34
Power:StatusTask	−2.58%	1.92%	−1.34	0.18
**Power:SNR**	**−0.49%**	**0.09%**	**−5.29**	**<1e-5**
StatusEO:SNR	0.11%	0.14%	0.79	0.43
StatusTask:SNR	0.24%	0.15%	1.53	0.13
Power:StatusEO:SNR	−0.09%	0.13%	−0.67	0.50
Power:StatusTask:SNR	0.07%	0.13%	0.55	0.59

Coefficients denote the change in EEG phase prediction accuracy as a percentage of 180°. For example, a +10% value would indicate that phase prediction accuracy increased by 10% of 180°, namely, by 18°. Significant predictors (*p* < 0.05) are shown in boldface.

### Effects on accuracy are robust to variations in 1/f and SNR computation pipelines

There has recently been an increase in different tools to compute the 1/f aperiodic background activity and SNR (Donoghue et al., 2020; Kosciessa et al., 2020; Whitten et al., 2011; Zrenner et al., 2020). The current analysis has used the approach of Zrenner et al. (2020), because of its simplicity and focus on real-time applications. However, this approach may not accurately fit the background noise, particularly in the presence of noise and nonlinearities, an issue which more modern approaches have tried to address.

We have replicated our analysis pipeline but have computed SNR over the whole recording using the extended Better Oscillation (eBOSC) pipeline (Kosciessa et al., 2020). eBOSC uses a robust regression along with time-frequency analysis using Morlet wavelets. Originally intended to identify periods of neural oscillations and characterize SNR over episodes, we have made slight modifications to compute a singular SNR value over the whole recording and have used these new values in our analyses without further changes. The individual alpha frequencies from eBOSC and Zrenner et al.’s (2020) approach showed a high correlation (*r* = 0.61, *p* < 1e-5), as well as the SNR values (*r* = 0.73, *p* < 1e-5). The main results remained the same, with a significant effect of power, SNR, and their interaction on accuracy.

### Effects on accuracy are robust to changes in data distribution

Unequal amounts of training data can potentially affect the prediction accuracies of the two conditions of interest. Although the linear mixed effects model is robust to unbalanced data (Schielzeth et al., 2020), the learned interpeak intervals from the ETP algorithm likely depends on the number of training samples. We ran a reanalysis, where both the number of included epochs for training and the average length of each epoch were kept consistent across all conditions. Observing that the number of tasks epochs was higher than the rest epochs, we removed task epochs uniformly across datasets, maintaining their relative contributions to the final model. Whereas we originally split resting-state data into two second epochs for both training and testing, we also decided to modify these lengths to match the averages of the task epochs. Our primary results remained the same, with a significant effect of power, SNR, and their interaction on accuracy. Additionally, there was a significant effect of power by task status by SNR (*t*_(9.29 *10^5^)_ = 2.31; *p* = 0.02), with an effect size of 0.31%, 95% CI [0.05%, 0.57%].

Despite matching the number of training epochs, the number of epochs used for testing was different because of additional artifact rejection criteria that was applied during testing. We performed an additional analysis where we matched the number of test epochs across the three conditions. The main results remained the same, with significant effects of power, SNR, and their interaction on accuracy. There were no additional significant results.

## Discussion

The current study examined whether different cognitive states affect EEG phase prediction accuracy and whether these differences are solely attributable to EEG instantaneous band power and SNR. Our primary hypothesis that rest states would show higher accuracy than task states was supported. Our base model, which did not include instantaneous power and SNR, indicated that the eyes-closed resting state had the highest accuracy followed by the eyes-open resting state and then the task state. It is worth noting that although the accuracy of the three states differed significantly, the accuracies were still close to each other (∼76% for EC rest and ∼74% for the task state). Our secondary hypothesis that all of the changes in prediction accuracy can be accounted for by SNR and band power differences were also supported. After accounting for power and SNR, we did not observe any differences in accuracy because of cognitive state. EEG power had a large and significant effect on EEG phase prediction accuracy. SNR also significantly affected phase prediction accuracy but with a small effect size. The effects of power and SNR showed a negative interaction, where an increase in one component led to a decrease in the effect of the other. However, our models indicated that including both covariates of power and SNR was necessary to explain the differences between cognitive states as some differences remained in prediction accuracy because of cognitive state when including only power.

We observed some unexpected patterns in band power between conditions in our analysis. Although the EC rest state had the highest average alpha power and highest accuracy, the EO rest state showed a lower average alpha power than the task states but a higher overall accuracy. This discrepancy is partially accounted for by the fact that the SNR of the EO rest state was almost double that of the task state. The higher SNR of the EO rest state also likely contributed to the significant interaction effect of the EO rest state with power in the intermediate model, which did not include SNR. The lower average alpha power of EO rest datasets was not isolated to a single dataset but was characteristic of the group; the three datasets with the lowest average alpha power were all EO rest datasets. It is counterintuitive that the task state had higher alpha power than the EO resting state, directly contradicting a previous study (Li, 2010) as well as prior findings that external attention should attenuate occipital alpha (van Dijk et al., 2008; Foxe and Snyder, 2011; Romei et al., 2010). Although it is true that external attention may attenuate alpha, there are several explanations why alpha power was higher in our task datasets. Participants may have experienced lapses of attention during parts of the task, which are associated with higher alpha power (Driel et al., 2012; Macdonald et al., 2011; Romei et al., 2010), or unmodeled environmental distractors and individual differences may have contributed to differing alpha powers (Aurlien et al., 2004; Hopman et al., 2020). Nevertheless, the resting-state datasets overall showed higher phase prediction accuracy than the task states. These differences were accounted for by band pass power and SNR; the differences in accuracy among conditions disappeared when including these covariates.

The rest states had almost double the SNR in the alpha band when compared with the task states. One potential explanation is that these increases in SNR were because of specific increases in alpha band power during the resting state. However, this reasoning would only apply to our eyes-closed resting-state data as the task state had higher average instantaneous power than the eyes-open resting state. Another explanation is that our SNR changes were because of changes in the overall power spectra, caused not just by the alpha band but by neighboring frequencies as well (Klimesch, 2018). For instance, Pathania et al. (2021) observed a steepening of the power spectra on task compared with rest. There is emerging evidence that the pattern in the power spectra depends on interindividual differences, task demands, and cognitive state (He, 2014; Henrie and Shapley, 2005; Klimesch, 2018; Miller et al., 2012). As a result, it is possible that the task datasets we analyzed revealed not only local increases in alpha, but also increases in other frequency bands that would have decreased the alpha SNR, yet still led to higher average instantaneous alpha power.

We also found a significant negative interaction between instantaneous alpha power and SNR, with power having a smaller effect on accuracy when SNR was higher. One explanation for this relationship could be a ceiling effect, where high SNR recordings already have a high baseline of accuracy, lessening the effect of power. The positive effects of power and SNR on accuracy, as well as the negative interaction between power and SNR, align with a prior investigation by Zrenner et al. (2020) into the practical limits of phase estimation methods. In their work, however, SNR was calculated on a per-epoch basis, whereas we calculated SNR on a per-recording basis. Consequently, our results show more variability for the same SNR value; even in low SNR recordings during periods of low alpha power, we saw some predictions yielding high accuracies. This result is likely if the prediction was in a high SNR epoch of a low SNR recording. Nevertheless, when averaged over the whole recording, our SNR effect sizes and directions align with prior results.

In one of our analyses where we equalized the number of training epochs used across conditions, we noticed a significant power by task by SNR interaction. We believe that this interaction is unique to the subset that we have chosen as we removed task epochs to match the number of rest epochs. We found this interaction only in this specific analysis and not in the other three analyses we have done. Two of the analyses were performed with approximately three times more data, indicating that this interaction likely does not generalize for larger samples.

Our results suggest that phase peaks can be consistently predicted across cognitive domains by targeting periods of high power and SNR. Experimenters do not need to induce a particular cognitive state to have high accuracy, at least in the case of occipital alpha, if they are willing to wait for periods of high power and SNR. The importance of signal power and quality is evidenced by the strong effect size of power and the general similarity of phase prediction accuracies between cognitive state conditions. The difference in the average phase prediction accuracy between our best and worst performing dataset was 5.75%, corresponding to a 10.35° deviation in phase. This difference is easily counteracted by waiting for periods of high power as a unit increase in power increases accuracy by 13.12%. Waiting for an appropriate period of high power should not take too long; the difference between the median and upper quartile of power in our datasets was over a unit increase in power, which should equal an average wait time of a few hundred milliseconds for an alpha wave.

These results suggest that phase–behavior studies should systematically consider the effects of power and SNR in their models. Although many current closed-loop BCI models do include a power criterion for stimulus presentation and stimulation (Vigué-Guix et al., 2022; Zrenner et al., 2018), their criteria are often different. For instance, Vigué-Guix et al. (2022) only considered windows that had an amplitude 30% above the median amplitude in the past 10 s, whereas Zrenner et al. (2018) manually adjusted amplitude thresholds to maintain a consistent firing rate. Finalized amplitude thresholds should be reported in all closed-loop studies to encourage replicability, a key issue in electrophysiological brain-behavior studies (Bigdely-Shamlo et al., 2015; Cohen, 2017). Finalized amplitude thresholds should be reported in all closed-loop studies to encourage replicability, a key issue in electrophysiological brain-behavior studies. Our current results suggest that a single amplitude threshold could suffice across cognitive domains as accuracy values across cognitive domains are similar at baseline and also because of a lack of an interaction between power and cognitive domain. In addition to closed-loop studies, open-loop studies should consider including power as a covariate. Most current phase–behavior studies use a measure of intertrial coherence (VanRullen, 2016), which does not depend on power. As EEG phase and power are intimately related (Zrenner et al., 2020), the use of amplitude-dependent measures of phase coherence may be more appropriate (Yoshinaga et al., 2020).

The current study used the ETP algorithm, which is notable for its efficiency in real-time contexts (Shirinpour et al., 2020), but the results could be expected to extend to other linear prediction techniques as the ETP algorithm shows similar performance to Fourier-based and autoregressive approaches (Mansouri et al., 2017; Shirinpour et al., 2020; Zrenner et al., 2018). Even in the current article, the phase prediction accuracy (mean offset of +6.30°, with SD of 47.34°) is comparable with previous attempts at phase targeting (Madsen et al., 2021; Vigué-Guix et al., 2022; Zrenner et al., 2020). It is likely that our results also extend to single-layered machine learning (ML) models of EEG phase prediction (McIntosh and Sajda, 2020), which are analogous to a linear regression. Interestingly, McIntosh and Sajda (2020) showed that these single-layered ML models performed similarly to more complex multilayered and gated-recurrent unit ML models. Nevertheless, investigation into the generalizability of our results to alternative algorithms is warranted. Of particular interest are dynamical systems approaches, such as state-space modeling (Matsuda and Komaki, 2017; Wodeyar et al., 2021), which have been shown to better track phase in situations involving broadband rhythms and phase resets.

We made several changes to the ETP algorithm to support epoched data analysis, which may affect the internal and external validity of some comparisons. Although Shirinpour et al. (2020) did not consider using epoched datasets for training, the training set was meant to be a representative sample from which to learn stable properties of the EEG waveform. Therefore, we do not expect much performance difference from using epoched than continuous data; although epoching may add more boundaries in the EEG recording and accordingly more edge filtering artifacts, the total length of data captured was like that of a continuous recording, and the ETP algorithm made sure to ignore samples near the edges. Another significant difference in the ETP implementation was that although the original algorithm learned the interpeak interval using pure resting-state recordings, we used intertrial intervals for the task states to learn interpeak intervals, as pure resting-state data were unavailable for many of our datasets. Significant neurocognitive adjustments may occur during intertrial intervals (Compton et al., 2011; King et al., 2010), potentially making the learned average interpeak interval inappropriate or at least not comparable with true rest. However, an exploratory analysis showed that training on these intertrial intervals provided the highest accuracy when compared with training on pure resting-state conditions, possibly because of shifts in alpha peak frequency that occur during different cognitive states (Mierau et al., 2017). Shirinpour et al.’s, (2020) study only included resting-state recordings in training and prediction; it is possible that training on the most similar cognitive state used for prediction would yield the highest accuracy, whether it is the resting state or the task state.

The fact that we can use intertrial intervals for training is significant. This interchangeability further emphasizes the stability of EEG phase across conditions. The results also suggest that phase-dependent BCI classifiers and other closed-loop machines can be effectively trained on intertrial intervals, in addition to distinct training sessions and rest periods. Including intertrial intervals could increase the amount of training data and statistical power available to BCI and research applications, as well as allow the phase-prediction model to adjust to any dynamic state changes throughout a recording.

Although we used datasets spanning multiple laboratories and countries, there was an imbalance among the represented conditions with task datasets making up 79.7% of all predictions. Also, certain datasets made up a large percentage of their group; one dataset made up 47.1% of the eyes-closed dataset. Future analyses can not only add more datasets but also make further refinements in dataset classification, specifying periods in a recording when different cognitive systems will be engaged, such as attention and memory. Researchers can also examine rest and task differences in a data-driven fashion, identifying power and topographic differences in different cognitive states across multiple frequency bands. A more hypothesis-driven approach is to use individualized spatial filters on hypothesized generators to get more accurate signals and track their movements in real time (Gordon et al., 2021). A particularly interesting covariate to include is the amount of nonstationarity in the EEG signal (Cao and Slobounov, 2011; Das and Nason, 2016), which makes signals harder to predict through nonsinusoidal distortions and phase resetting (Matsuda and Komaki, 2017; Wodeyar et al., 2021). Using these phase-prediction models, we could identify good and bad periods of phase prediction accuracy, giving greater insight into the cognitive and neurobiological correlates of EEG phase. Future studies, in both research and clinical settings, could use these results to optimize EEG phase prediction accuracy in closed-loop and BCI implementations to administer more effective interventions and stimuli.

## Conclusion

Our results indicate that existing methods can track EEG phase accurately across various cognitive conditions and datasets and that accuracy can be enhanced by waiting for high periods of instantaneous band power and SNR. Our base model demonstrated that cognitive state (eyes closed, eyes open, task) affects EEG phase prediction accuracy, with the eyes-closed resting state being the most accurate, followed by the eyes-open resting state, then by the task state. Nevertheless, the absolute accuracy differences were relatively small and attributable to EEG power and SNR. Accordingly, experiments, closed-loop technologies, and BCIs implementing real-time EEG phase prediction should use protocols that minimize the influence of external unwanted noise and target periods of high power for maximum accuracy as opposed to manipulating experimental and cognitive conditions. We also showed that phase prediction models can be trained on intertrial intervals for prediction during on-task periods. Future studies and BCI implementations may benefit from using a similar approach to obtain a higher number of trials and a more dynamic model. Additional research into other frequency bands and sites of interests and covariates such as nonstationarities are warranted to understand the cognitive and neurobiological correlates of EEG phase and how they inform the optimization of EEG phase prediction accuracy in closed-loop applications and BCIs.

## Synthesis

Reviewing Editor: Niko Busch, Westfalische Wilhelms-Universitat Munster Decisions are customarily a result of the Reviewing Editor and the peer reviewers coming together and discussing their recommendations until a consensus is reached. When revisions are invited, a fact-based synthesis statement explaining their decision and outlining what is needed to prepare a revision will be listed below. The following reviewer(s) agreed to reveal their identity: Andreas Wutz, Irene Vigué-Guix. Note: If this manuscript was transferred from JNeurosci and a decision was made to accept the manuscript without peer review, a brief statement to this effect will instead be what is listed below.

# Synthesis

Your manuscript has been reviewed by two experts on EEG-based BCI and phase analysis. Both reviews are largely positive and highlight the merit of your study. However, the reviewers have a few requests for further clarifications. Among the larger points are a request for a reanalysis balancing the size of datasets in the resting state vs. on-task conditions, and for contextualizing the study more in terms of the extant literature. I encourage you to carefully address each of the reviewers’ points.

# Reviewer 1

This paper compares the accuracy of an EEG-phase prediction algorithm, namely the Educated Temporal Prediction (ETP) algorithm, across 11 different datasets and 543 participants. The main aim was to investigate the phase prediction accuracy for alpha-frequency oscillations between different cognitive states (i.e. eyes-open and eyes-closed resting state, and on-task state) while also taking into account the impact of overall alpha power and the signal-to-noise ratio (SNR). The results showed better prediction accuracies for resting state vs. on-task data, which however, were in large parts due to differences in power and SNR.

In my view, the paper is clearly and concisely written and the included Figures depict the technical approach quite nicely. At large, the employed EEG methodology and the analytical and statistical aspects are sound. Moreover, the substantial body of processed data (11 data sets, > 500 subjects) constitute a particular asset of the paper. The results are clear and support the authors’ final conclusions. Nevertheless, I do have one major point of criticism alongside some more minor issues, which need to be addressed, before I can give a final recommendation.

Major:

The main contrast of their analysis refers to the comparison between resting state vs. on-task conditions in terms of phase prediction accuracy. However from the description on pp. 16/17 (ll. 300 ff), it seems that an unequal amount of data went into the algorithm training and testing between resting state vs. on-task conditions. While the authors made sure that data within each condition was equally split between training and testing (which I think is not strictly necessary), there was an imbalance between the conditions of interest (i.e. on-task, eyes-open and eyes-closed resting state) In my eyes, this necessarily should bias prediction accuracies between the two conditions of interest. Consequently, I would like to see a re-analysis, in which the amount of training and testing data was equal between resting state and on-task conditions. This refers to the number of processed trials, as well as to the distributions of included temporal interval durations.

Minor:

1. The Title and the Abstract somewhat contradict each other. Whereas in the Title you write that there is an effect of cognitive state on prediction accuracy, in the Abstract you write that there is none. I do understand that this effect is mediated by SNR and power, and that this is also reflected in the Title, but when reading it at first glance the statements appear inconsistent.

2. I would highly appreciate, if you could included some more descriptive data figures. For example, one figure could show the used EEG sensors. Another useful figure could show a distribution of the extracted individual alpha peaks. Moreover, I was not able to make out whether the waveforms were extracted from each single used parieto-occipital electrode an then combined after prediction or whether the waveform was averaged first over electrodes and then used for prediction.

3. It would be useful to know what the prediction accuracies meant in terms of time (in ms) or phase angles. How close along the alpha cycle were the predicted values?

4. For the nested model comparison, I would advise to increase the model complexity step-wise from the basic model to an intermediate model that only includes one moderating factor (i.e. power or SNR) and the up to a full model, in order to see whether a simpler model could already satisfactorily explain the effects.

5. In my view, the second formula on p. 18 (l. 350) is formally incorrect.

For example, “state + state * SNR ...” would mean that the factors “state + state + SNR + their element-wise product” was included. So “state” would be in there twice. I believe that you entered the factors correctly into your model and that only the notion here is incorrect, but please clarify this aspect for me. Please see for comparison: https://www.mathworks.com/help/stats/fitlme.html

6. About the first formula on p. 18 (l. 334): What is the point of “dataset_id : individual_id”, if each subject contributed only on dataset each. Isn’t this redundant then? Please clarify.

On a related note, I did read that there was one dataset with 36 participants, which contained both resting state and on-task data. In this case, the sentence on p. 18, l. 340 “each dataset and individual were part of only a rest or task dataset” would not be correct, right? Importantly, however, this one dataset with data from both conditions of interest would make for an excellent add-on analysis, in which the reported effects with respect to training on ITI vs. training on rest could be compared within participants.

# Reviewer 2

MAJOR

1. Regarding the overall standpoint of the manuscript, I refer the authors to some important previous empirical and theoretical literature that is not currently considered in the manuscript and it’s relevant to the research field.

- In the intro, there is a brief section on how the alpha phase has been linked to different cognitive functions. However, the role of the alpha phase in cognition is still under debate, and I would suggest approaching the brain-behavior link more cautiously including citations of some papers against the evidence (e.g., see Ruzzoli et al., 2019; Keitel et al., 2022).

- There is a key paper missing in the manuscript that could support the study in many aspects: an empirical study that presented stimuli in a visual perception task depending on the alpha phase using a real-time closed-loop EEG-based BCI (Vigué-Guix et al., 2020). It appears that the main ideas, methods, and results of the study are in line with the current results, and this literature should be cited. It would be interesting to hear the author’s thoughts on how their study contributes to this line of research.

- In the intro, when talking about the background activity, I would suggest reading and including the work of Peterson and Voytek (2017), Lundqvist & Wutz, (2021), and Iemi et al. (2022).

2. Could the authors provide citations to give support to the following ideas appearing in the manuscript?

- In the intro, when saying “Better phase estimation improves targeting accuracy, decreases statistical variance, and consequently increases the potential customizability and effect size of interventions (REF/s)”.

- In the methods, when saying “The algorithm requires no assumptions about the underlying signal and has been shown to perform more quickly and accurately than the other approaches (REF/s).”

- In the methods, properly cite Delorme & Makeig (2004) after EEGLAB and add the citation for MATLAB (Natick, Massachusetts: The MathWorks Inc).

- In the methods, for the individual alpha frequency (IAF). Many studies have recently used the concept but is designated differently. Which method have you used?

3. Questions for the authors:

- Could the authors provide citations/arguments of why they chose the criteria for removing the channels (e.g., why 4 using SDs and not 3; have others used this criterion?)? The way they are written looks kind of arbitrary.

- Regarding the IAF (lines 272-273), what happens if a participant in a given trial had two or three peaks in the alpha band? I would suggest the authors be more specific on the criteria and include the factor of uniqueness besides the strong peak (highest power). See Ruzzoli et al. (2019) and Vigué-Guix et al. (2020) as examples.

- I found inconsistencies in the alpha-band across the manuscript: in the preprocessing the band-pass filter goes from 8 to 13 Hz and in the designation of the IAF is from 8 to 14 Hz. Why don’t you keep it simple and use the same? It’s confusing.

- Why the authors have computed the 1/f noise and the SNR the way they did and didn’t use other currently available and more robust methods (e.g., BOSC by Whitten et al., 2011 or eBOSC by Kosciessa et al., 2020)?

- Could the authors provide more information about why they choose the bands to perform the fitting? If they want to remove the alpha band from the fitting, why do they fit between 0.5 - 7 Hz and 35-65 Hz, and not 0.5 - 7 Hz and 13-65 Hz?

- Have the authors considered that eyes-closed and eyes-open conditions could be associated with different types of alpha activity (generators)? (see Lundqvist & Wutz, 2021).

- It could be insightful if the authors provide a comparison of their phase prediction accuracy (10.35º) with the accuracy of other studies (e.g., Zrenner et al. 2018; Vigué-Guix et al, 2020). It would lead to a discussion of whether the methods they used were the best and whether other phase prediction methods (such as the ones used in the mentioned papers) would be a better fit (or not). Please, note that if one would target a specific phase in the alpha-cycle using a closed-loop BCI (such as in Vigué-Guix’s study) 10º is noticeably.

MINOR

- Avoid repeating twice “sending and receiving” in lines 40-42

- Could the authors provide the exact timing in parenthesis of the “brief delay” in the task section (line 175)?

- In the “Educated Temporal Prediction Algorithm” section, it should be resourceful to add another potential way to predict phase using the Hilbert transform by extending a sinus wave from a reference point in the EEG signal (see Vigué-Guix, 2020).

- Suggestion of adding “(see EEG preprocessing section)” in lines 202-203.

- Add punctuation to help read the criteria for removing channels (in lines 158-261).

- The dataset link of Zanesco et al. (2020) leads to a ‘404 error’.

- Correct for general missing spaces across the manuscript (e.g., line 289).

- Bold format in lines 377-378

- Could the authors be consistent when presenting the p-values in the results section?

- Writing typo in lines 407

- The link provided in line 566 leads to a ‘404 error’.

REFERENCES

Iemi, L., Gwilliams, L., Samaha, J., Auksztulewicz, R., Cycowicz, Y. M., King, J.‑R., Nikulin, V. V., Thesen, T., Doyle, W., Devinsky, O., Schroeder, C. E., Melloni, L., & Haegens, S. (2022). Ongoing neural oscillations influence behavior and sensory representations by suppressing neuronal excitability. NeuroImage, 247, 118746. https://doi.org/10.1016/j.neuroimage.2021.118746.

Lundqvist, M., & Wutz, A. (2021). New methods for oscillation analyses push new theories of discrete cognition. Psychophysiology, e13827. https://doi.org/10.1111/psyp.13827

Keitel, C., Ruzzoli, M., Dugué, L., Busch, N. A., & Benwell, C. S. Y. (2022). Rhythms in cognition: The evidence revisited. European Journal of Neuroscience, 55(11-12), 2991-3009. https://doi.org/10.1111/ejn.15740

Kosciessa, J. Q., Grandy, T. H., Garrett, D. D., & Werkle-Bergner, M. (2020). Single-trial characterization of neural rhythms: Potential and challenges. NeuroImage, 206, 116331. https://doi.org/10.1016/j.neuroimage.2019.116331

Peterson, E. J., & Voytek, B. (2017). Alpha oscillations control cortical gain by modulating excitatory-inhibitory background activity. https://doi.org/10.1101/185074

Ruzzoli, M., Torralba, M., Morís Fernandez, L., & Soto-Faraco, S. (2019). The relevance of alpha phase in human perception. Cortex, 120, 249-268. https://doi.org/10.1016/j.cortex.2019.05.012

Vigué-Guix, I., Fernandez, L. M., Cuello, M. T., Ruzzoli, M., & Soto-Faraco, S. (2020). Can the occipital alpha-phase speed up visual detection through a real-time EEG-based brain-computer interface (BCI)? European Journal of Neuroscience, 55(11-12), 3224-3240. https://doi.org/10.1111/ejn.14931

Whitten, T. A., Hughes, A. M., Dickson, C. T., & Caplan, J. B. (2011). A better oscillation detection method robustly extracts EEG rhythms across brain state changes: The human alpha rhythm as a test case. NeuroImage, 54(2), 860-874. https://doi.org/10.1016/j.neuroimage.2010.08.064

## Author Response

# Reviewer 1

This paper compares the accuracy of an EEG-phase prediction algorithm, namely the Educated Temporal Prediction (ETP) algorithm, across 11 different datasets and 543 participants. The main aim was to investigate the phase prediction accuracy for alpha-frequency oscillations between different cognitive states (i.e. eyes-open and eyes-closed resting state, and on-task state) while also taking into account the impact of overall alpha power and the signal-to-noise ratio (SNR). The results showed better prediction accuracies for resting state vs. on-task data, which however, were in large parts due to differences in power and SNR. In my view, the paper is clearly and concisely written and the included Figures depict the technical approach quite nicely. At large, the employed EEG methodology and the analytical and statistical aspects are sound. Moreover, the substantial body of processed data (11 data sets, > 500 subjects) constitute a particular asset of the paper. The results are clear and support the authors’ final conclusions. Nevertheless, I do have one major point of criticism alongside some more minor issues, which need to be addressed, before I can give a final recommendation.

- Response:

We thank the reviewer for their positive remarks and have worked to thoroughly address their critiques.

Major:

The main contrast of their analysis refers to the comparison between resting state vs. on-task conditions in terms of phase prediction accuracy. However from the description on pp. 16/17 (ll. 300 ff), it seems that an unequal amount of data went into the algorithm training and testing between resting state vs. on-task conditions. While the authors made sure that data within each condition was equally split between training and testing (which I think is not strictly necessary), there was an imbalance between the conditions of interest (i.e. on-task, eyes-open and eyes-closed resting state) In my eyes, this necessarily should bias prediction accuracies between the two conditions of interest. Consequently, I would like to see a re-analysis, in which the amount of training and testing data was equal between resting state and on-task conditions. This refers to the number of processed trials, as well as to the distributions of included temporal interval durations. 2

- Response:

We thank the reviewer for their comment. We agree that unequal amounts of training data can potentially affect the prediction accuracies across conditions of interest. Although the linear mixed effects model is robust to unbalanced data (Schielzeth et al., 2020), the learned interpeak intervals from the Educated Temporal Prediction algorithm likely depend on the number of training samples entered.

We have done a re-analysis where the amount of data in the training and testing dataset was equalized, based on the number of trials as well as the average duration lengths. Generally speaking, all of the main effects of power, SNR, and the interaction between power and SNR were found to be significant in the re-analyses. In one of the analyses, however, we found an additional task by power by SNR interaction. We believe this interaction is likely a result of sampling error because it does not appear in any of the three other analyses and has a relatively small effect size.

We have added a paragraph in our Methods section (page 24) describing the issue of imbalanced data and the approach that we took to solve it: “Unequal amounts of training data can potentially affect the prediction accuracies of the two conditions of interest. Although the linear mixed effects model is robust to unbalanced data (Schielzeth et al., 2020), the learned interpeak intervals from the ETP algorithm likely depends on the number of training samples. We ran a re-analysis, where both the number of included epochs for training and the average length of each epoch were kept consistent across all conditions. Observing that the number of tasks epochs was higher than the rest epochs, we removed task epochs uniformly across datasets, maintaining their relative contributions to the final model. Whereas we originally split resting-state data into two second epochs for both training and testing, we also decided to modify these lengths to match the averages of the task epochs. Our primary results remained the same, with a significant effect of power, SNR, and their interaction on accuracy. Additionally, there was a significant effect of power by task status by SNR (t(9.29 *105 ) = 2.31; p = 0.02), with an effect size of 0.31%, 95% CI [0.05%, 0.57%]. Despite matching the number of training epochs, the number of epochs used for testing was different due to additional artifact rejection criteria that happens during testing. We performed an additional analysis where we matched the number of test epochs across the three conditions. The main results remained the same, with significant effects of power, SNR, and their interaction on accuracy. There were no additional significant results.”

We have also included a paragraph in the discussion section (page 27) discussing the significant task by power by SNR interaction and why we believe that it would not generalize beyond that subset: ”In one of our analyses where we equalized the number of training epochs used across conditions, we noticed a significant power by task by SNR interaction. We believe that this interaction is unique to the subset that we have chosen, as we removed task epochs to match the number of rest epochs. We found this interaction only in this specific analysis 3 and not in the other three other analyses we have done. Two of the analyses were performed with approximately three times more data, indicating that this interaction likely does not generalize for larger samples.”

Minor:

1. The Title and the Abstract somewhat contradict each other. Whereas in the Title you write that there is an effect of cognitive state on prediction accuracy, in the Abstract you write that there is none. I do understand that this effect is mediated by SNR and power, and that this is also reflected in the Title, but when reading it at first glance the statements appear inconsistent.

- Response:

We thank the reviewer for their comment. We agree that the Title and Abstract seem contradictory. We have modified the Title to emphasize the absence of a difference after accounting for power and SNR as follows: “EEG Phase Can Be Predicted With Similar Accuracy Across Cognitive States After Accounting For Power and SNR”

2. I would highly appreciate, if you could included some more descriptive data figures. For example, one figure could show the used EEG sensors. Another useful figure could show a distribution of the extracted individual alpha peaks. Moreover, I was not able to make out whether the waveforms were extracted from each single used parieto-occipital electrode an then combined after prediction or whether the waveform was averaged first over electrodes and then used for prediction.

- Response:

We thank the reviewer for their comment. We agree that more descriptive data figures would increase the readability of the current manuscript. To address this issue, we have included the following figure showing the electrode of interest as well as the surrounding electrodes used for the spatial filtering. Figure 2. EEG Electrode Placement and Surface Laplacian. a) Diagram of the 10-10 system used in the current analysis. The electrodes utilized in the Laplacian montage are colored with the central electrode (Pz) in red, and the surrounding electrodes (Oz, Cz, P4, and P3) in yellow. b, c) Applying the Laplacian montage allows us to see higher spatial frequencies. Scalp topographies in the alpha band (8-13 Hz) are displayed without (b) and with (c) the Laplacian montage applied. 4 The spatial filtering / averaging was applied prior to ETP algorithm. We have added the following to our methods section (page 14): “The ETP algorithm also applies spatial filtering via the Laplacian montage (R. Gordon & Rzempoluck, 2004), using this spatially filtered signal for prediction. For our Laplacian montage, we chose to center on electrode Pz, with surrounding electrodes Oz, Cz, P4 and P3, as parieto-occipital electrodes exhibit the highest alpha power (Figure 2).”

We have also included the following figure describing some of the summary statistics of all the datasets, including the distribution of accuracy, alpha peaks, power, and SNR. Figure 3. Accuracy, Signal-to-noise ratio (SNR), Individual Alpha Frequency (IAF), and Instantaneous Power Distributions across datasets. The height of each bar represents the average value while the error bars represent one standard deviation. Bars are filled based on which cognitive state they represent. Abbreviations are used for each dataset name, refer to Table 1 for the full dataset name and references.

3. It would be useful to know what the prediction accuracies meant in terms of time (in ms) or phase angles. How close along the alpha cycle were the predicted values?

- Response:

We thank the reviewer for their comment. We agree that including other metrics besides accuracy may make the results clearer. We have updated the Results section (Page 20) to also include phase angle deviations and time, and how they relate to previously reported values: “Across all of the predictions made by the algorithm on the datasets, the average phase prediction accuracy was 73.70% (SD = 23.40%). However, accuracy only describes the spread of the predictions. In terms of phase angles, our algorithm was on average +6.30{degree sign} away from the target (SD = 47.34{degree sign}). In the context of a 10Hz alpha wave, our predictions were 3.5ms late (SD = 13.15ms). These results align with previous attempts at phase-targeting (Madsen et al., 2019; Vigué-Guix et al., 2022; Zrenner et al., 2018), whose procedures have hit the target from -12{degree sign} to 5{degree sign}, with standard deviations from 25{degree sign} to 55{degree sign}.” 5 4. For the nested model comparison, I would advise to increase the model complexity step-wise from the basic model to an intermediate model that only includes one moderating factor (i.e. power or SNR) and the up to a full model, in order to see whether a simpler model could already satisfactorily explain the effects. - Response:

We thank the reviewer for their comment. We agree that increasing the model step-wise may help show the separate contributions of each factor transparently. We have updated the Methods section (Page 19) by adding the following section: “We used the following intermediate model to identify the effects of rest or task on phase prediction accuracy with alpha power as a covariate: accuracy ∼ state + alpha_power + state:alpha_power + (alpha_power | dataset_id) + (alpha_power | dataset_id : individual_id) “ We have also updated the Results section (Page 21) to include the intermediate model with one moderating factor of power. “When including just power as a covariate, the baseline accuracy of EC rest dropped to 64.94%, 95% CI [63.4%, 66.4%]. Power had a significant effect on accuracy (t(1.51*106 ) = 9.98; p < 1e-5). A unit increase in instantaneous power led to a 6.80% increase in accuracy, 95% CI [5.46%, 8.13%]. After accounting for power, the EO rest state did not significantly affect accuracy ( t(1.51*106 ) = -1.66; p = 0.10). However, there was a significant interaction of the EO rest state and power ( t(1.51*106 ) = 2.17; p = 0.03), with an effect size of 2.06%, 95% CI [0.20%, 3.92%]. After accounting for power, the task state still had a significant effect on accuracy (t(1.51*106 ) = -2.17; p = 0.03), with an effect size of -2.27%, 95% CI [-0.22%, -4.32%]. However, there was no significant interaction between the task state and power ( t(1.51*106 ) = 0.52; p = 0.60). These results do not align with our hypothesis that differences in phase prediction accuracy due to cognitive states do not persist after accounting for instantaneous power. A likelihood ratio test indicated that this model explained the data better than the prior model (χ²(20) = 9182, p < 1e-5).” As power alone does not explain all of the results, we have updated the Discussion section (Page 25) as follows: “After accounting for power and SNR, we did not observe any differences in accuracy due to cognitive state. EEG power had a large and significant effect on EEG phase prediction accuracy. SNR also significantly affected phase prediction accuracy, but with a small effect size. The effects of power and SNR showed a negative interaction, where an increase in one component led to a decrease in the effect of the other. However, our models indicated that including both covariates of power and SNR was necessary to explain the differences between cognitive states, as some differences remained in prediction accuracy due to cognitive state when including only power.” 6

5. In my view, the second formula on p. 18 (l. 350) is formally incorrect.

For example, “state + state * SNR ...” would mean that the factors “state + state + SNR + their element-wise product” was included. So “state” would be in there twice. I believe that you entered the factors correctly into your model and that only the notion here is incorrect, but please clarify this aspect for me. Please see for comparison: https://www.mathworks.com/help/stats/fitlme.html

- Response:

We thank the reviewer for their comment. We agree that when expanded formally, the formula would have a duplicate term of state. For correctness, we have changed the syntax to “state:SNR”, which does not include the lower-order terms, to the methods section (Page 20) and have verified that the results do not change with this new formula: “accuracy ∼ state + state : SNR + state : alpha_power + state : alpha_power : SNR + (alpha_power : SNR | dataset_id) + (alpha_power : SNR | dataset_id : individual_id)”

6. About the first formula on p. 18 (l. 334): What is the point of “dataset_id : individual_id”, if each subject contributed only on dataset each. Isn’t this redundant then? Please clarify.

- Response:

We thank the reviewer for their comment. The reviewer is correct that for the current analysis, each individual was part of a single dataset. The reason “dataset_id : individual_id” is included is due to a practical matter of ID coding. Although individuals are not shared across datasets, their ID‘s are shared. Specifically, the first individual in each dataset was given an individual_id of 1, and then 2, and so on. The nested random effect of “dataset_id : individual_id” allows the model to distinguish an individual with the id of 1 as belonging to a particular dataset. If ID’s were unique across the whole analysis (1 to 543), then the “dataset_id : individual_id” would indeed be redundant. To avoid this confusion for future readers, we have added the following sentences in the Methods section (Page 19) to make this point clearer: “The nested random effect of dataset_id : individual_id was included due to how we coded our variables. Specifically, the first individual in each dataset was given an individual_id of 1, and then 2, and so on. The nested random effect of dataset_id : individual_id allowed us to distinguish an individual with the id of 1 as belonging to a particular dataset.”

On a related note, I did read that there was one dataset with 36 participants, which contained both resting state and on-task data. In this case, the sentence on p. 18, l. 340 “each dataset and individual were part of only a rest or task dataset” would not be correct, right? Importantly, however, this one dataset with data from both conditions of interest would make for an excellent add-on analysis, in which the reported effects with respect to training on ITI vs. training on rest could be compared within participants.

- Response:

7

We thank the reviewer for their comment. The reviewer is correct that one dataset had participants in both resting and on-task data (Wong et al., 2018). For this special dataset, we decided to model the resting and on-task portions as separate datasets with different individuals for consistency and efficiency, as it was our only dataset that had both conditions and adding the random intercepts would significantly increase the complexity of the model. We have updated the paragraph in the methods section (Page 19) to make this distinction clearer: “Although there was a single dataset that contained both rest and task data (Wong et al., 2018), we decided to model the rest and task data as distinct datasets with different individual. This simplification was made to promote consistency with the other datasets that only had recordings of a single type and to reduce the complexity of our linear models.”

We agree that this dataset offers many opportunities for add-on analyses. We have completed a additional analysis of the differential effects of training on resting-state versus inter-trial intervals for task-data prediction. We observed that training on ITIs led to the highest accuracy, potentially due to the higher similarity of ITIs with task data than rest. We have mentioned these results in the discussion section (page 29): “Another significant difference in the ETP implementation was that while the original algorithm learned the interpeak interval using pure resting-state recordings, we used inter-trial intervals for the task states to learn interpeak intervals, as pure resting-state data was unavailable for many of our datasets. Significant neurocognitive adjustments may occur during inter-trial intervals (Compton et al., 2011; King et al., 2010), potentially making the learned average interpeak interval inappropriate, or at least not comparable to true rest. However, an exploratory analysis showed that training on these inter-trial intervals provided the highest accuracy when compared to training on pure resting-state conditions, possibly due to shifts in alpha peak frequency that occur during different cognitive states (Mierau et al., 2017). Shirinpour et al.‘s study only included resting-state recordings in training and prediction (Shirinpour et al., 2020), it is possible that training on the most similar cognitive state used for prediction would yield the highest accuracy, whether it is the resting-state or the task-state.”

# Reviewer 2

MAJOR

1. Regarding the overall standpoint of the manuscript, I refer the authors to some important previous empirical and theoretical literature that is not currently considered in the manuscript and it’s relevant to the research field.

- In the intro, there is a brief section on how the alpha phase has been linked to different cognitive functions. However, the role of the alpha phase in cognition is still under debate, and I would suggest approaching the brain-behavior link more cautiously including citations of some papers against the evidence (e.g., see Ruzzoli et al., 2019; Keitel et al., 2022).

- Response: We thank the reviewer for their comments. We agree that we should have approached the brain-behavior link more cautiously as there is published evidence that scalp EEG detected phase- 8 behavior relationships are equivocal. To address this issue, we have changed the introduction section to be more cautious with regards to the brain-behavior links including citations of papers against the evidence and have mentioned the importance of more rigor in these experiments.

We have made the following changes on page 3: “It is important to note that EEG phase-behavior relationships are still under question as recent replication attempts have shown some null results (Keitel et al., 2022; Ruzzoli et al., 2019). Further understanding of the potential links between EEG phases and behaviors will need to come from both theoretical and empirical studies. Theoretically, postulating how brain rhythms are generated and interact with each other within the broader neurobiological context will be critical. Empirically, EEG phase-behavior experiments need stronger and more robust causal demonstrations. Closed-loop BCIs are an attractive candidate for these demonstrations due to the strong limitations they impose on phase-behavior experiments. As behavior is manipulated in real-time, a closed-loop BCI must choose their pre-processing parameters and features of interest beforehand. Furthermore, closed-loop BCI experiments allow for precise control of stimulus presentation based on brain states, allowing us to draw stronger causal inferences in EEG phase-behavior relationships (Ramot & Martin, 2022; Vigué-Guix et al., 2022).” - There is a key paper missing in the manuscript that could support the study in many aspects: an empirical study that presented stimuli in a visual perception task depending on the alpha phase using a real-time closed-loop EEG-based BCI (Vigué-Guix et al., 2020). It appears that the main ideas, methods, and results of the study are in line with the current results, and this literature should be cited. It would be interesting to hear the author’s thoughts on how their study contributes to this line of research.

- Response:

We thank the reviewer for their comment. We agree that the study of Vigué-Guix and colleagues is relevant to the current paper, as it uses a closed-loop BCI to target alpha phase in a speeded reaction-time task. Their paper is situated in the broader context of showing brain-behavior relationships which, as mentioned in the previous comment, has mixed results. We think that their paper addresses concerns regarding rigor but still believe that a lack of standardization is an overarching concern in this literature. We included the paper as a reference as described in our response to the previous comment, where we elaborated on how closed-loop BCIs can allow for stronger empirical demonstrations of the phase-behavior relationship. We also expanded upon the latter point of inconsistent methodology while referencing the closed-loop BCI of ViguéGuix in the discussion section.

We have added the following section on page 28: “These results suggest that phase-behavior studies should systematically consider the effects of power and SNR in their models. Although many current closed-loop BCI models do include a power criterion for stimulus presentation and stimulation (ViguéGuix et al., 2022; Zrenner et al., 2018), their criteria are often different. For instance, 9

Vigué-Guix and colleagues only considered windows that had an amplitude 30% above the median amplitude in the past ten seconds, while Zrenner and colleagues manually adjusted amplitude thresholds to maintain a consistent firing rate. Finalized amplitude thresholds should be reported in all closed-loop studies to encourage replicability, a key issue in electrophysiological brain-behavior studies (Bigdely-Shamlo et al., 2015; M. X. Cohen, 2017). Our current results suggest that a single amplitude threshold could suffice across cognitive domains, as accuracy values across cognitive domains are similar at baseline and also due to a lack of an interaction between power and cognitive domain. In addition to closed-loop studies, open-loop studies should consider including power as a covariate. Most current phase-behavior studies use a measure of inter-trial coherence (VanRullen, 2016), which does not depend on power. As EEG phase and power are intimately related (Zrenner et al., 2020), the use of amplitude dependent measures of phase coherence may be more appropriate (Yoshinaga et al., 2020).”

- In the intro, when talking about the background activity, I would suggest reading and including the work of Peterson and Voytek (2017), Lundqvist & Wutz, (2021), and Iemi et al. (2022).

- Response:

We thank the reviewer for these helpful references. We agree that all the provided references can help support our section of background activity. We previously suggested that cognitive states can affect either the frequency band of interest or the background noise but have neglected the interaction between the two. The provided references help tie these two processes together, indicating how the power of alpha oscillations are related to both inhibitory/excitatory background and high frequency activity. We have included them when discussing the background activity section on page 4: “Cognitive states can affect EEG phase prediction accuracy either through direct changes in the frequency of interest (Zrenner et al., 2020), or through indirect changes in the surrounding background noise (He, 2014; Pathania et al., 2021). Furthermore, these two factors are linked, with direct changes in one frequency band, specifically alpha, causing changes in the background excitatory, inhibitory, and high frequency activity (Iemi et al., 2022; Peterson & Voytek, 2017). There is also evidence that different thalamic and cortical alpha generators are differentially activated and coupled under different task conditions (Halgren et al., 2019; Lundqvist & Wutz, 2022; Saalmann et al., 2012). These highly dynamic changes in EEG activity necessitate more flexible analysis tools that can account for these changes, such as phase or burst analysis (Lundqvist & Wutz, 2022). However, these tools can be further improved by directly incorporating the dynamics of cognitive state.”

2. Could the authors provide citations to give support to the following ideas appearing in the manuscript?

- In the intro, when saying “Better phase estimation improves targeting accuracy, decreases statistical variance, and consequently increases the potential customizability and effect size of interventions (REF/s)”. 10

- Response:

We thank the reviewer for their comment. We agree that the statement needs some references to fully justify its argument. We have re-worded the sentence (page 3) as follows to focus more on the variability/precision of phase estimation, which directly relates to statistical power, while also including references: “Real-time phase-behavior studies and phase-targeted interventions depend on accurate and precise instantaneous phase estimates. Better phase estimation improves targeting accuracy and precision, which decreases statistical variance and directly increases the statistical power of these studies (J. Cohen, 2013).” - In the methods, when saying “The algorithm requires no assumptions about the underlying signal and has been shown to perform more quickly and accurately than the other approaches (REF/s).”

- Response:

We thank the reviewer for their comment. We agree that this statement needs a reference and have neglected to include the reference to the original paper by Shirinpour et al. (2020). In this paper, the researchers do a direct comparison of the ETP algorithm with Fourier Transform and Autoregressive approaches and shows the algorithm’s strong performance in terms of accuracy and computational speed. As for the claim that the algorithm requires no assumptions, we have scaled back the statement on page 13 to “minimal assumptions”. - In the methods, properly cite Delorme & Makeig (2004) after EEGLAB and add the citation for MATLAB (Natick, Massachusetts: The MathWorks Inc). - Response:

We thank the reviewer for this helpful reminder and have included these citations in the appropriate locations.

- In the methods, for the individual alpha frequency (IAF). Many studies have recently used the concept but is designated differently. Which method have you used?

- Response:

We thank the reviewer for their question. We have utilized the method used in Zrenner et al. (2020). The basic method is computing the peak in the power spectra after subtracting the 1/f noise, which was computed through linear regression in the log-log spectrum. We decided to use this approach for its conceptual and methodological simplicity. Furthermore, Zrenner et al. were focused on testing real-time phase estimation in the context of closed-loop BCI (2020). We added the following portion to the methods section (page 16): 11 “To quantitatively model differences in signal quality across recordings, we decided to include SNR in our statistical models. We used the approach of Zrenner et al. in computing SNR (2020), due to its simplicity and focus on real-time phase estimation.”

3. Questions for the authors:

- Could the authors provide citations/arguments of why they chose the criteria for removing the channels (e.g., why 4 using SDs and not 3; have others used this criterion?)? The way they are written looks kind of arbitrary.

- Response:

We thank the reviewer for the question. We have utilized the default settings from the “clean_rawdata” plugin from EEGLAB, which uses methods from the BCILAB Toolbox (Kothe & Makeig, 2013). We agree that 4 standard deviations may seem high, but it has precedent in the BCI/EEG literature that use this plugin (Chang et al., 2020). Furthermore, the standardized early stage EEG processing pipeline (PREP) utilizes a criteria of 5 standard deviations (Bidgely-Shamlo et al., 2015), indicating that 4 standard deviations may not be too high. To alleviate readers’ concerns, we have included the following sentence in the methods section (page 15): “While 4 standard deviations above the population mean is aggressive, it is consistent with the default settings of the clean_rawdata plugin, and has precedence in the BCI literature and other preprocessing pipelines which use 4 or 5 standard deviations (Bigdely-Shamlo et al., 2015; Chang et al., 2020).” - Regarding the IAF (lines 272-273), what happens if a participant in a given trial had two or three peaks in the alpha band? I would suggest the authors be more specific on the criteria and include the factor of uniqueness besides the strong peak (highest power). See Ruzzoli et al. (2019) and Vigué-Guix et al. (2020) as examples.

- Response:

We thank the reviewer for their question. If there were more than one peak in the alpha band, the IAF was chosen as the frequency with the highest SNR (defined as distance from power spectrum to the 1/f line). We were more concerned with the presence of an alpha oscillation, so we excluded recordings without a local peak. We do agree that this measure may be inadequate when there are multiple peaks within the alpha band. Whereas Vigué-Guix et al. have rejected epochs with multiple peaks, we decided to go with the approach of Klimesch (1999), who computed the weighted sum of the peak frequencies and their powers. We re-did the analysis with this new condition and added the following sentence to the methods section appropriately (page 16): “In the case of more than one spectral peak in the 8 and 13Hz range, we designated the IAF as the average of the peak frequencies weighted by their alpha power (Klimesch, 1999).” 12

- I found inconsistencies in the alpha-band across the manuscript: in the preprocessing the band pass filter goes from 8 to 13 Hz and in the designation of the IAF is from 8 to 14 Hz. Why don’t you keep it simple and use the same? It’s confusing. - Response: We thank the reviewer for the helpful suggestion. We have updated the designation of the IAF from 8 to 13 Hz to remain consistent throughout the processing pipeline. - Why the authors have computed the 1/f noise and the SNR the way they did and didn’t use other currently available and more robust methods (e.g., BOSC by Whitten et al., 2011 or eBOSC by Kosciessa et al., 2020)?

- Response:

We thank the reviewer for their comment. We agree that BOSC and eBOSC offer more robust methods of calculating the 1/f noise and SNR compared to Zrenner’s method. eBOSC in particular may give different values as it uses a robust regression to down-weight spectral peaks. Furthermore, instead of using Welch’s method to compute the frequency spectra, (e)BOSC uses wavelet analysis to compute the time-frequency plot and then takes the average as needed to compute the frequency spectra. However, BOSC and eBOSC have different use cases than Zrenner’s method, making the latter method more appropriate for us. We wanted to use SNR as a summary statistic capturing the overall signal quality of a recording. Zrenner’s approach easily allows this as the 1/f curve and SNR is computed over the whole recording. On the other hand, BOSC and eBOSC only calculates these metrics for identified periods of significant oscillations based on a power and duration threshold. These identified episodes may not align with the pseudo-rest and task epochs that we have created, leading to difficulties in calculating a summary statistic of SNR for the whole recording, and even for a single epoch of interest. Nevertheless, we believe it is possible to modify the eBOSC algorithm to compute a single SNR value over the whole recording, by taking the average of the time-frequency plot. Doing so would allow us to leverage the robust regression and wavelet analysis that eBOSC performs, while also giving us an appropriate summary statistic that we can use to characterize the quality of each recording.

We have run an additional analysis with the modified eBOSC algorithm, and have added the following portion to the Results Section (page 23): “There has recently been an increase in different tools to compute the 1/f aperiodic background activity and SNR (Donoghue et al., 2020; Kosciessa et al., 2020; Whitten et al., 2011; Zrenner et al., 2020). The current analysis has utilized the approach of Zrenner et al. (2020), due to its simplicity and focus on real-time applications. However, their approach may not accurately fit the background noise, particularly in the presence of noise and non-linearities, an issue which more modern approaches have tried to address. 13

We have replicated our analysis pipeline but have computed SNR over the whole recording using the extended Better Oscillation (eBOSC) pipeline (Kosciessa et al., 2020). eBOSC utilizes a robust regression along with time-frequency analysis using Morlet wavelets. Originally intended to identify periods of neural oscillations and characterize SNR over episodes, we have made slight modifications to compute a singular SNR value over the whole recording and have used these new values in our analyses without further changes. The individual alpha frequencies from eBOSC and Zrenner’s approach showed a high correlation (r = 0.61, p < 1e-5), as well as the SNR values (r = 0.73, p < 1e-5). The main results remained the same, with a significant effect of power, SNR, and their interaction on accuracy.”

- Could the authors provide more information about why they choose the bands to perform the fitting? If they want to remove the alpha band from the fitting, why do they fit between 0.5 - 7 Hz and 35-65 Hz, and not 0.5 - 7 Hz and 13-65 Hz?

- Response:

We thank the reviewer for their question. We agree with the reviewer about the potential confusion about the fitting process. This process was the same one carried out by Zrenner et al. (2020), who justified the decision as fitting the line at “fixed frequencies outside of known oscillations”. Zrenner et al. were interested in the mu rhythm, which contains both alpha and beta components. However, since we are only interested in the alpha band, we have changed the fit to be based off of 0.5 - 8 Hz and 13 - 65 Hz.

- Have the authors considered that eyes-closed and eyes-open conditions could be associated with different types of alpha activity (generators)? (see Lundqvist & Wutz, 2021).

- Response:

We thank the reviewer pointing out the omission of relevant information regarding the nature of alpha generators. We agree that there is evidence of different alpha generators depending on cognitive states, which may better inform our introduction and discussion section. Namely, the differences in alpha generators add to the initial reasons why we might expect differences in prediction accuracies in the two states. Likewise, one possible future direction is adding more accurate and individualized spatial filters to extract alpha signals from these generators (Gordon et al., 2021). We have added the following portion to the introduction section (page 4): “Cognitive states can affect EEG phase prediction accuracy either through direct changes in the frequency of interest (Zrenner et al., 2020), or through indirect changes in the surrounding background noise (He, 2014; Pathania et al., 2021). Furthermore, these two factors are linked, with direct changes in one frequency band, specifically alpha, causing changes in the background excitatory, inhibitory, and high frequency activity (Iemi et al., 2022; Peterson & Voytek, 2017). There is also evidence that different thalamic and cortical alpha generators are differentially activated and coupled under different task conditions (Halgren et al., 2019; Lundqvist & Wutz, 2022; Saalmann et al., 2012). These highly dynamic changes in EEG activity necessitate more flexible analysis tools that can account for these changes, such as phase or burst analysis (Lundqvist & 14 Wutz, 2022). However, these tools can be further improved by directly incorporating the dynamics of cognitive state.”

We have added the following section to the discussion section in the future directions paragraph (page 30): “Researchers can also examine rest and task differences in a data-driven fashion, identifying power and topographic differences in different cognitive states across multiple frequency bands. A more hypothesis-driven approach is to utilize individualized spatial filters on hypothesized generators to get more accurate signals and track their movements in real-time (P. Gordon et al., 2021).” - It could be insightful if the authors provide a comparison of their phase prediction accuracy (10.35º) with the accuracy of other studies (e.g., Zrenner et al. 2018; Vigué-Guix et al, 2020). It would lead to a discussion of whether the methods they used were the best and whether other phase prediction methods (such as the ones used in the mentioned papers) would be a better fit (or not). Please, note that if one would target a specific phase in the alpha-cycle using a closed-loop BCI (such as in Vigué-Guix’s study) 10º is noticeably.

- Response:

We thank the reviewer for their comment. We agree with the reviewer comparing the accuracy values from ETP to the other algorithms can help situate the paper in its broader context and help with future directions. We have included the following section in the results section (page 20) when we include phase prediction accuracy: “Across all of the predictions made by the algorithm on the datasets, the average phase prediction accuracy was 73.70% (SD = 23.40%). However, accuracy only describes the spread of the predictions. In terms of phase angles, our algorithm was on average +6.30{degree sign} away from the target (SD = 47.34{degree sign}), while in the context of a 10Hz alpha wave, our predictions were 3.5ms late (SD = 13.15ms). These results align at previous attempts at phase-targeting (Madsen et al., 2019; Vigué-Guix et al., 2022; Zrenner et al., 2018), whose procedures had a mean error of -12{degree sign} to 5{degree sign}, with standard deviations from 25{degree sign} to 55{degree sign}.”

MINOR

- Avoid repeating twice “sending and receiving” in lines 40-42

- Response:

We thank the reviewer for their suggestion and have fixed the wording.

- Could the authors provide the exact timing in parenthesis of the “brief delay” in the task section

(line 175)?

- Response:

We thank the reviewer for their suggestion and have added the delay.

15

- In the “Educated Temporal Prediction Algorithm” section, it should be resourceful to add another potential way to predict phase using the Hilbert transform by extending a sinus wave from a reference point in the EEG signal (see Vigué-Guix, 2020).

- Response:

We thank the reviewer for their suggestion. We have added the following comparison between the ETP method to Vigué-Guix et al.’s method in the methods section (page 13): “A very similar approach has been used by Vigué-Guix et al. in a real-time closed-loop BCI context that performed similarly to other estimate approaches (2022). The main difference in their approach was basing predictions off of the individual alpha frequency instead of the average interpeak interval, the distance between consecutive peaks.”

- Suggestion of adding “(see EEG preprocessing section)” in lines 202-203.

- Response:

We thank the reviewer for their suggestion and have added the comment.

- Add punctuation to help read the criteria for removing channels (in lines 158-261).

- Response:

We thank the reviewer for their suggestion and have added commas while listing out the criteria for removing channels.

- The dataset link of Zanesco et al. (2020) leads to a ‘404 error’.

- Response:

We thank the reviewer for their suggestion. We have checked that the dataset link works on multiple web browsers. We have attached the hyperlink here for your reference: https://ftp.gwdg.de/pub/misc/MPI-Leipzig_Mind-Brain-Body-LEMON/

- Correct for general missing spaces across the manuscript (e.g., line 289).

- Response:

We thank the reviewer for their correction. We have checked the original manuscript and observed correct spacing at line 289. We believe there might be an issue with the formatting when merging on the journal side. We will keep an eye out for this issue on future submissions.

- Bold format in lines 377-378

- Response:

We thank the reviewer for the suggestion and have added bold format for sentences indicating how the results relate to our hypotheses. 16

- Could the authors be consistent when presenting the p-values in the results section?

- Response:

We thank the reviewer for the suggestion and have modified the presentation of p-values to be more consistent.

- Writing typo in lines 407

- Response:

We thank the reviewer for the correction and have fixed the typo.

- The link provided in line 566 leads to a ‘404 error’.

- Response:

We thank the reviewer for their comment and have fixed the link:
